# It Takes Two to Tango: Combining Conventional Culture With Molecular Diagnostics Enhances Accuracy of *Streptococcus pneumoniae* Detection and Pneumococcal Serogroup/Serotype Determination in Carriage

**DOI:** 10.3389/fmicb.2022.859736

**Published:** 2022-04-18

**Authors:** Willem R. Miellet, Janieke van Veldhuizen, David Litt, Rob Mariman, Alienke J. Wijmenga-Monsuur, Paul Badoux, Tessa Nieuwenhuijsen, Rebecca Thombre, Sanaa Mayet, Seyi Eletu, Carmen Sheppard, Marianne Alice van Houten, Nynke Y. Rots, Elizabeth Miller, Norman K. Fry, Elisabeth A. M. Sanders, Krzysztof Trzciński

**Affiliations:** ^1^Department of Pediatric Immunology and Infectious Diseases, Wilhelmina Children’s Hospital, University Medical Center Utrecht (UMCU), Utrecht, Netherlands; ^2^Centre for Infectious Disease Control, National Institute for Public Health and the Environment (RIVM), Bilthoven, Netherlands; ^3^Respiratory and Vaccine Preventable Bacterial Reference Unit (RVPBRU), Public Health England – National Infection Service, London, United Kingdom; ^4^Regional Laboratory of Public Health (Streeklab) Haarlem, Haarlem, Netherlands; ^5^Spaarne Gasthuis, Haarlem, Netherlands; ^6^Immunisation and Countermeasures Division, Public Health England (PHE) – National Infection Service, London, United Kingdom

**Keywords:** *Streptococcus pneumoniae* (pneumococcus), carriage, diagnostic accuracy, qPCR (quantitative PCR), conventional culture

## Abstract

**Background:**

The specificity of molecular methods for the detection of *Streptococcus pneumoniae* carriage is under debate. We propose a procedure for carriage surveillance and vaccine impact studies that increases the accuracy of molecular detection of live pneumococci in polymicrobial respiratory samples.

**Methods:**

Culture and qPCR methods were applied to detect pneumococcus and pneumococcal serotypes in 1,549 nasopharyngeal samples collected in the Netherlands (*n* = 972) and England (*n* = 577) from 946 toddlers and 603 adults, and in paired oropharyngeal samples collected exclusively from 319 Dutch adults. Samples with no live pneumococci isolated at primary diagnostic culture yet generating signal specific for pneumococcus in qPCRs were re-examined with a second, qPCR-guided culture. Optimal C_q_ cut-offs for positivity in qPCRs were determined via receiver operating characteristic (ROC) curve analysis using isolation of live pneumococci from the primary and qPCR-guided cultures as reference.

**Results:**

Detection of pneumococcus and pneumococcal serotypes with qPCRs in cultured (culture-enriched) nasopharyngeal samples exhibited near-perfect agreement with conventional culture (Cohen’s kappa: 0.95). Molecular methods displayed increased sensitivity of detection for multiple serotype carriage, and implementation of qPCR-guided culturing significantly increased the proportion of nasopharyngeal and oropharyngeal samples from which live pneumococcus was recovered (*p* < 0.0001). For paired nasopharyngeal and oropharyngeal samples from adults none of the methods applied to a single sample type exhibited good agreement with results for primary and qPCR-guided nasopharyngeal and oropharyngeal cultures combined (Cohens kappa; 0.13–0.55). However, molecular detection of pneumococcus displayed increased sensitivity with culture-enriched oropharyngeal samples when compared with either nasopharyngeal or oropharyngeal primary cultures (*p* < 0.05).

**Conclusion:**

The accuracy of pneumococcal carriage surveillance can be greatly improved by complementing conventional culture with qPCR and *vice versa*, by using results of conventional and qPCR-guided cultures to interpret qPCR data. The specificity of molecular methods for the detection of live pneumococci can be enhanced by incorporating statistical procedures based on ROC curve analysis. The procedure we propose for future carriage surveillance and vaccine impact studies improves detection of pneumococcal carriage in adults in particular and enhances the specificity of serotype carriage detection.

## Introduction

*Streptococcus pneumoniae* (pneumococcus) is the most common etiological agent of invasive bacterial disease (O’Brien et al., 2009) and of community-acquired pneumonia of bacterial etiology (Welte et al., 2012). Despite being vaccine-preventable, pneumococcal disease remains among the leading causes of death in childhood (World Health Organization, 2019) as available vaccines target only a various subsets of only 24 from ca. 100 of the known serotypes. Due to high pneumococcal carriage rates (Bogaert et al., 2004; Satzke et al., 2013) children are considered the primary reservoir of pneumococcus and the main drivers of transmission and infections in any population (Wyllie et al., 2016a,^2020^; Flasche et al., 2020). Children are also the primary group targeted with pneumococcal vaccination (Berical et al., 2016).

With pneumococcal vaccines protecting not only against disease but also against colonization, carriage is now an accepted endpoint in vaccination studies (Dagan et al., 1997; van Gils et al., 2009; Flasche et al., 2011; Auranen et al., 2013). Following the introduction of pneumococcal conjugate vaccines (PCV), of which 10-valent (PHiD-CV, GSK), 13-valent (Prevnar 13, Pfizer), 15-valent (Vaxneuvance, MSD), and 20-valent (Prevnar 20, Pfizer) are currently marketed, epidemiological surveillance of carriage became an essential tool for monitoring the direct and indirect (herd protection) effects of vaccination. Surveillance of carriage is also used to detect the emergence and track expansion of non-vaccine serotypes, the phenomenon described as vaccine-induced serotype replacement (Weinberger et al., 2011). Finally, carriage studies are instrumental in monitoring serotype-associated invasiveness (Sleeman et al., 2006).

To understand the trends in pneumococcal epidemiology and guide strategies for PCV use, it is critical to establish methodology for *S. pneumoniae* carriage detection that is sufficiently sensitive across all ages (Slotved, 2016; Weinberger et al., 2016). However, conventional culture, which is considered to be the gold standard method in carriage detection, is not suitable for detection of multiple serotype carriage (Valente et al., 2013) and lacks the sensitivity when used to detect *S. pneumoniae* in age groups other than children (Krone et al., 2014). There is also evidence that testing solely a single site within the upper airways reduces sensitivity of carriage detection (Korona-Glowniak et al., 2011; Trzcinski et al., 2013; Almeida et al., 2020).

Molecular methods have largely improved the sensitivity of *S. pneumoniae* detection and have made multiple serotype carriage detection feasible (Turner et al., 2011; Satzke et al., 2013). Using assays developed by our groups and others, we have tested the serotype composition of respiratory samples from children and adults and have demonstrated an under-detection of *S. pneumoniae* and of individual pneumococcal serotypes by culture when compared with molecular methods (Trzcinski et al., 2013; Wyllie et al., 2014, 2016a,b; Krone et al., 2015; Miellet et al., 2020). However, some caution that molecular methods exhibit poor specificity due to the presence of pneumococcal genes among commensal streptococci (Carvalho Mda et al., 2012, 2013; Boelsen et al., 2020). It could also be argued that molecular detection is unable to discriminate between live bacteria and presence of relic DNA (Lennon et al., 2018).

Here, we outline the protocol that combines conventional culturing with pneumococcus-specific qPCRs and employs statistical procedures to interpret the molecular results and enhances the specificity of the molecular methods. We show that molecular methods applied to nasopharyngeal samples demonstrate near-perfect agreement with primary culture and yet the sensitivity of *S. pneumoniae* carriage surveillance can be greatly enhanced by complementing conventional culture with qPCRs and *vice versa*. We also show that testing nasopharyngeal samples alone leads to underestimation of pneumococcal carriage in adults.

## Materials and Methods

### Study Design and Ethics Statement

Pneumococcal carriage was investigated in two cross-sectional prospective observational studies conducted in 2015/2016 in community-dwelling individuals in the Netherlands (Vissers et al., 2018) and in England (Southern et al., 2018). The study conducted in the Netherlands was approved by the Medical Ethics Committee Noord Holland (NTR5405 on).^1^ The study conducted in England was approved by the NHS Health Research Authority and the London Fulham Research Ethics Committee (reference 15/LO/0458) and was registered on clinicaltrials.gov (reference NCT02522546). In both studies written informed consent was obtained from the parent or guardian of every participating child and adults provided written consent for their own participation. Both studies were conducted in accordance with Good Clinical Practice and the Declaration of Helsinki.

### Sample Collection and Laboratory Processing

Respiratory samples were collected in the Netherlands between October 2015 and March 2016 in the study on carriage of respiratory bacterial pathogens coordinated by National Institute of Public Health and the Environment. Nasopharyngeal samples were collected in children aged 24 months (±1 month) vaccinated with PHiD-CV according to “2 primary + 1 booster” (2p + 1b) dose schedule, children aged 44–49 months vaccinated with PHiD-CV in 3p + 1b schedule, and parents of the 24-month-old children (one parent per child). Oropharyngeal swabs were also collected from Dutch adults (Watt et al., 2004; Vissers et al., 2018). The English study took place between July 2015 and June 2016 and was conducted by the National Vaccine Evaluation Consortium which included Public Health England (Southern et al., 2018). In England, nasopharyngeal samples were collected in children aged 1–5 years vaccinated with PCV13 according to a 2p + 1b schedule and in their household contacts (adults aged > 20 years).

All samples were obtained according to World Health Organization standard procedures (Satzke et al., 2013). Immediately after sampling, swabs in the Netherlands were placed in liquid Amies transport medium (ESwab 482C, Copan, Brescia, Italy) and within 8 h transported to the diagnostic laboratory. In England swabs were placed in skim-milk, tryptone, glucose and glycerol (STGG) broth and delivered to a diagnostic laboratory within 48 h (Table 1).

**TABLE 1:** Methodological differences between *Streptococcus pneumoniae* carriage studies conducted in the Netherlands and in England.

Processing step	The Netherlands	England
Transport medium	1 ml Amies	1 ml STGG
Transport temperature	Ambient	2–8°C
Duration of transport	<8 h	<48 h
Arrival to the diagnostic lab	Culturing on receipt	Storage at −80°C
Culture medium	BA-GENT and CBA	COBA and CBA
Inoculum volume	35 μl	50 μl
Identification of *S. pneumoniae*	Optochin-susceptibility and bile solubility tests	Bioinformatics-based method applied to bacterial DNA
Minimally processed (MP) sample	An aliquot of Amies stored at −80°C	An aliquot of skim milk tryptone glucose and glycerine broth medium (STGG) stored at −80°C
Culture-enriched (CE) sample	Whole BA-GENT plate colony growth washed with 2.1 ml of BHI with 10%glycerol	Swab of α-hemolytic colonies from COBA (first choice) or CBA, and into 1 ml of STGG

*STGG, skim milk tryptone glucose and glycerine broth medium; CBA, Columbia blood agar plate; BA-GENT, blood agar plate gentamycin; COBA, blood agar plate with Streptococcus selective medium.*

### Conventional Culture

Thirty-five microliters (Netherlands) or 50 μl (England) of specimen was used as an inoculum in detection of pneumococci by conventional culture (primary culture). In the Netherlands samples were cultured on SB7-Gent agar selective for streptococci (BA-GENT, Oxoid, Badhoeve Dorp, The Netherlands) and on non-selective Columbia blood agar (CBA, Oxoid). In England samples were cultured on Streptococcus-selective Blood Agar (COBA, Oxoid, Basinstoke, United Kingdom) and CBA (Oxoid). Remaining transport media were stored frozen at −70°C. In the Netherlands, samples were aliquoted into 200 μl volumes, and a single aliquot was supplemented with glycerol (10% v/v final concentration) prior to freezing.

After overnight incubation at 37°C and 5% CO_2_, cultures were screened for pneumococcus-like colonies to be re-cultured. Once screened, in the Netherlands all colony growth was harvested from BA-GENT plate into 10% glycerol in brain heart infusion broth (Oxoid) (Miellet et al., 2020). In England, all colony growth was harvested from any plates containing alpha-hemolytic colonies into PBS, centrifuged, the supernatant removed, and any growth stored as a pellet (Southern et al., 2018). These samples were considered culture-enriched (CE) for pneumococci and stored at −80°C. Cultured strains (one per sample, but more if distinct pneumococcal morphotypes were apparent) were serotyped by Quellung method in the Netherlands (Vissers et al., 2018) and with the previously described PneumoCaT bioinformatic pipeline in England that was supplemented with slide agglutination when required (Kapatai et al., 2016; Southern et al., 2018). Since non-typeable thus unencapsulated pneumococci are not the target of pneumococcal vaccines and are also avirulent, cultured non-typeable strains were not considered in the analysis. Non-typeable pneumococci are negative for *piaB* (Whalan et al., 2006; Trzcinski et al., 2013; Wyllie et al., 2017) and their inclusion would complicate a comparison of detection methods.

### Molecular Detection of *Streptococcus pneumoniae*

Molecular detection of *S. pneumoniae* in respiratory samples was conducted as described previously (Miellet et al., 2020). For this, Amies medium, STGG medium, and culture-enriched samples were shipped on dry ice from primary diagnostic sites to the study central laboratory in the Netherlands. There, pellets of culture-enriched samples collected in England were reconstituted to the 200 μl that matched original volume of an aliquot. Next, nucleic acids were extracted from 100 μl of the transport media using the DNeasy Blood and Tissue Kit (Qiagen) and eluted into 200 μl. These extractions represented minimally processed samples. For culture-enriched samples, 100 μl of a bacterial growth harvest was centrifuged for 2 min at 14,000 × g, the pellet was resuspended with 90 μl of TE buffer [20 mM Tris-HCl (pH 8.0), 2 mM EDTA] and incubated for 15 min at 95°C. Next, 90 μl of lysis buffer [20 mM Tris-HCl (pH 8.0), 2 mM EDTA, 2.4% Triton X-100 and 40 mg/ml lysozyme] was added, and the samples were processed as above. Pneumococcal DNA was detected via single plex qPCR (Supplementary Table 1) using primers and probe targeting sequences (Supplementary Table 2) within genes coding for the pneumococcal iron uptake ABC transporter lipoprotein PiaB (Trzcinski et al., 2013), and for the major pneumococcal autolysin LytA (Carvalho Mda et al., 2007) by testing 5.5 μl DNA from minimally processed or 1.0 μl of culture-enriched samples within a total qPCR reaction volume of 12.5 μl.

### Molecular Detection of Pneumococcal Serotypes

DNA extracted from culture-enriched harvests was used to determine the serotype composition of nasopharyngeal and oropharyngeal samples using a panel of primers and probes (Azzari et al., 2010, 2012; Pimenta et al., 2013), targeting all serotypes covered by pneumococcal vaccines available either in the Netherlands or in England at the time of sample collection (PHiD-CV, PCV13 and 23-valent polysaccharide vaccine, PPV23, Merck Sharp Dohme) except for serotype 2. The panel also covered selected non-vaccine serotypes (serotypes 6C, 6D, 7A, 10B, 12A, 12B, 15A, 15C, 15F, 16F, 21, 22A, 23A, 23B, 33A, 34, 35B, 35F, 37, and 38). However, with certain assays targeting more than one serotype it was not possible to distinguish between serotypes 6A and 6B; 6C and 6D; 7A and 7F; 9A, 9L, 9N and 9V; 10A and 10B; 11A and 11D; 12A, 12B and 12F; 15A, 15B, 15C and 15F; 33A, 33F and 37; 35B and 35C when detected with qPCR. A sample pooling strategy was employed when testing for serotypes in order to reduce the number of serotype-specific qPCRs. For this, samples generating any signal below C_q_ 40 for either *piaB* or *lytA* were pooled in groups of five to be tested (Wyllie et al., 2016a) and the remaining samples were pooled in groups of ten. Samples negative for *piaB* and *lytA* were tested to assess specificity of serotype/serogroup-specific assays (Wyllie et al., 2016a). Samples from pools generating a signal for a particular serotype/serogroup were tested individually. In the Netherlands, the qPCRs were performed on the LightCycler 2 platform and in England they were performed on the QuantStudio 7 platform, using identical PCR conditions.

### qPCR-Guided Recovery of Live *Streptococcus pneumoniae* Strains

Culture-enriched samples classified as negative in primary diagnostic culture but positive by qPCR were revisited with culture in a second attempt to isolate live pneumococci. For this, CBA plates were inoculated with 100 μl of 10^–1^–10^–3^ and 10^–3^–10^–5^ dilution of culture-enriched nasopharyngeal and culture-enriched oropharyngeal samples, respectively, and incubated at 37°C and 5% CO_2_. Pneumococcus-like colonies were individually tested in qPCR for *piaB* and *lytA* and confirmed to be *S. pneumoniae* based on susceptibility to optochin.

### Statistical Analysis

Data analysis was performed in GraphPad Prism software version 9.1.0 and R version 4.1.0. Receiver operating characteristic (ROC) curve analysis was performed using the “cutpointr” R package to validate qPCR results with culture results (primary culture plus qPCR-guided culture). Maximum Youden index values, the sum of sensitivity and specificity minus one, were estimated via bootstrapping (*n* = 1,000) on *piaB* and *lytA* qPCR data from respiratory samples to determine optimal cut-off values for qPCR detection (Nutz et al., 2011). Two-way mixed effects intraclass correlations (ICCs) (Koo and Li, 2016) and Bland-Altman plots (Bland and Altman, 1986) were used to evaluate agreement for quantitative results using the “ïrr” and “blandr” R packages, respectively. Carriage rates were compared with McNemar’s test unless otherwise stated. Estimates for accuracy of diagnostic tests between methods (subgroups) were compared with a test of interaction (Altman and Bland, 2003). A *p*-value of < 0.05 was considered significant. Diagnostic test parameters (predictive values, sensitivity, and specificity) were calculated using an in-house made R function,^2^ and 95% confidence intervals were calculated with Wilson-Brown score. Cohen’s kappa, a measure of chance-corrected agreement, and its 95% confidence interval was calculated as described by McHugh (2012) and ratios of ≤ 0, 0.01–0.20, 0.21–0.40, 0.41–0.60, 0.61–0.80, and > 0.81 were interpreted as exhibiting poor, slight, fair, moderate, substantial, and near-perfect agreement, respectively (Landis and Koch, 1977). For comparison between serotyping by culture and by qPCR, analysis was limited to qPCR-targeted serotypes. For serogroup-specific qPCR assays a result was considered congruent when a serogroup was detected in qPCR that matched the serogroup of the serotype detected by culture. Expected frequencies of multiple serotype carriage were calculated squaring observed pneumococcal prevalence rates and expected and observed multiple serotype frequencies were compared using a one-proportion *Z*-test.

### Assessment of Method’s Inter-Laboratory Reproducibility

One-hundred seventy-six nasopharyngeal samples collected in England and *n* = 59 oropharyngeal samples collected in the Netherlands have been selected to evaluate the inter-laboratory reproducibility of molecular methods. For this, an aliquot of minimally processed STGG nasopharyngeal sample, a fresh preparation of culture enriched nasopharyngeal sample from a subset of the English study samples and culture-enriched oropharyngeal samples from a subset of the Dutch study samples were tested at the study site in England, as described above. Results for paired samples were compared between centers by calculating the percent agreement and Cohen’s kappa. Quantitative results of both laboratories were also compared by calculating an intraclass correlation coefficient (ICC) and by comparing results in Bland-Altman plots. Carriage rates between both laboratories were compared using Cohen’s kappa.

## Results

**FIGURE 1:**
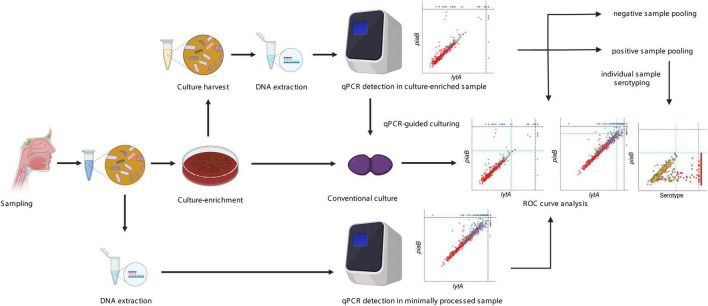
Overview of the “two-to-tango” protocol. A respiratory sample is collected from a study participant and 200 μl sample is split into two parts. One part is used directly for DNA extraction while another part is plated on selective culture media for culture-enrichment. Detection of *S. pneumoniae* presence by conventional culture is conducted on the culture-enriched sample. Once completed, all microbial growth is harvested in a broth supplemented with glycerol. DNA extraction is performed on the raw sample and culture harvest. Molecular detection of *S. pneumoniae* is conducted with *piaB* and *lytA* qPCRs and culture-negative but qPCR positive samples are revisited for qPCR-guided culturing. Receiver operating characteristic curve analysis is performed to increase the specificity of qPCR detection for presence of live pneumococcus. A sample pooling strategy is conducted, samples negative for pneumococcus are pooled by 10 and samples positive for pneumococcus are pooled by five. Serotyping by qPCR is conducted on pooled samples. If the pool is classified as positive all samples are tested individually for a given serotype. Negative pools are used to evaluate the specificity of serotype-specific qPCR assays. The figure was made using Biorender.

We evaluated the performance of conventional and molecular methods in a new protocol (Figure 1) and applied it to detect *S. pneumoniae* and pneumococcal serotypes in 1,549 nasopharyngeal samples collected from 946 children aged 1–5 years (*n* = 653 in the Netherlands and *n* = 293 in England) and from 603 adults (*n* = 319 in the Netherlands and *n* = 284 in England), and on oropharyngeal samples from 319 adults in the Netherlands. All qPCR results shown were generated by testing the Dutch and English samples in the Dutch laboratory, except when stated otherwise.

### Detection of *Streptococcus pneumoniae*

**FIGURE 2:**
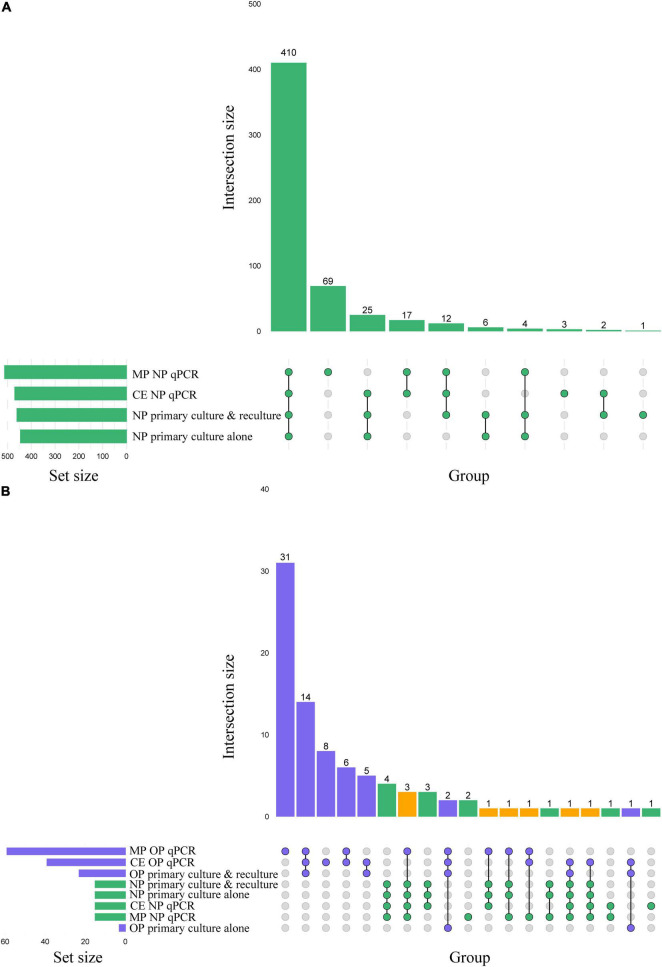
**(A)** Matrix layout for observed intersections of the *Streptococcus pneumoniae* detection procedures applied to *n* = 1,549 nasopharyngeal (NP) samples, sorted by size. **(B)** Matrix layout for observed intersections of the *S. pneumoniae* detection procedures applied to nasopharyngeal and oropharyngeal (OP) samples from *n* = 319 adults sampled in the Netherlands, sorted by size. Circles in the matrix indicate sets that are part of the intersection, with nasopharyngeal and oropharyngeal samples colored in green and blue, respectively. The bar diagram displaying the intersection size is colored using the same scheme, with orange indicating an intersection that represents positivity in both sample types. MP stands for minimally processed and CE for culture-enriched.

When analyzing culturing results without the addition of qPCR-guided culturing, live *S. pneumoniae* was isolated from 29% (445/1,549) of primary cultures of nasopharyngeal swabs (Figure 2A), with significantly higher fraction of samples from children (45% or 422/946) compared with adults (4% or 23/603) positive for pneumococcus (Pearson’s chi-square, *p* < 0.0001). Among 319 adults sampled in the Netherlands (Figure 2B), live pneumococcus was significantly more often cultured from nasopharyngeal (*n* = 15 or 5%) compared with oropharyngeal (*n* = 3 or 1%) samples (*p* < 0.01).

**FIGURE 3:**
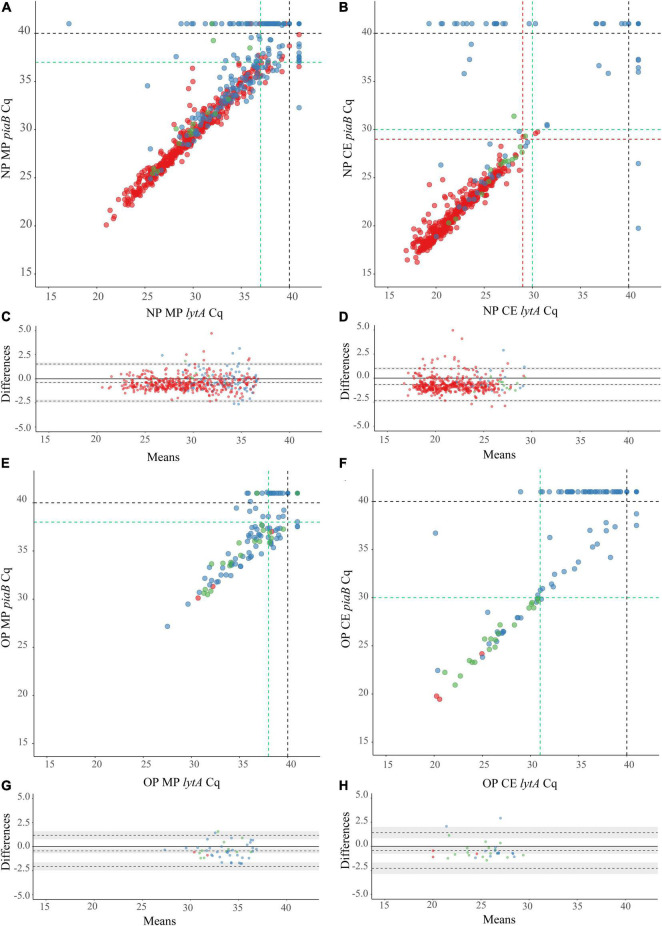
Scatter plots **(A,B,E,F)** displaying correlation between cycle threshold (C_q_) from qPCR assays targeting the *Streptococcus pneumoniae* genes *piaB* and *lytA* for minimally processed **(A,E)** and culture-enriched **(B,F)** nasopharyngeal **(A,B)** and oropharyngeal **(E,F)** samples followed by the Bland-Altman plots **(C,D,G,H)** displaying agreement between *piaB* and *lytA* for samples positive with minimally processed **(C,G)** and culture-enriched **(D,H)** ^ROCd^C_q_ criteria in nasopharyngeal **(C,D)** and oropharyngeal **(G,H)** samples. The shaded gray areas in **(G,H)** mark the 95% confidence interval of the upper limit of agreement, bias and lower limit of agreement. The Bland-Altman plots show the absence of systematic bias between *piaB* and *lytA* because the line of equality (solid line) is within the limits of agreement. Bias between *piaB* and *lytA* is more often observed in high C_q_ samples. Primary culture-positive and qPCR-guided culture-positive samples are indicated with red and green dots, respectively. Culture-negative samples are indicated with blue dots. In **(A,B,E,F)** black dashed lines indicate the arbitrary C_q_ criterium (<40 C_q_), green dashed lines indicate ^ROCd^C_q_ criteria derived by validating qPCR results with primary and qPCR-guided cultures results. Red dashed lines in **(B)** indicate ^ROCd^C_q_ criteria derived by validating qPCR results with primary culture alone. In **(A)** red lines overlap with green lines and only the latter are shown.

Next, we conducted qPCRs on minimally processed and culture-enriched samples. To enhance the specificity of detection we used a “two-to-tango” approach by quantifying *piaB* and *lytA* genes and considering a sample to be positive for *S. pneumoniae* when both targets were detected. When applying an arbitrary quantification cycle (^A^C_q_) of < 40 C_q_ as criterium for positivity altogether 583 nasopharyngeal swabs (38% of 1,549) were identified as positive for pneumococcus (Figures 3A,B). The fraction of positive samples was significantly higher among minimally processed compared with culture-enriched swabs (561 or 36% vs. 479 or 31% of 1,549; *p* < 0.0001). In line with results of primary diagnostic culture, here too the proportion of positive samples was higher in children compared with adults (Pearson’s chi-square, *p* < 0.0001) whenever nasopharyngeal swabs were tested minimally processed (56% or 528/946 vs. 5% or 33/603) or culture-enriched (48% or 454/946 vs. 4% or 25/603). However, contrary to results of primary diagnostic culture, among adults significantly larger proportions of oropharyngeal compared with nasopharyngeal samples (*p* < 0.0001) have been classified as positive for pneumococcus with qPCR whenever tested minimally processed (26% or 83/319 vs. 6% or 19/319) or culture-enriched (18% or 58/319 vs. 6% or 18/319).

Next, samples negative for pneumococcus in primary diagnostic cultures yet generating signal in qPCRs for *piaB* and *lytA* were cultured again. This qPCR-guided culture effort increased the number of samples from which live pneumococci was cultured, in relatively small albeit significant increase of 3% (from 445 vs. 460 of 1,549; *p* < 0.0001) for nasopharyngeal (Figures 2A, 3A,B) and 666% increase (from 3 to 23 of 319; *p* < 0.0001) for oropharyngeal samples (Figures 2B, 3E,F).

To validate qPCR results using culture and to further enhance the specificity of qPCR methods for the detection of live pneumococci we performed receiver operating characteristic (ROC) curve analysis and identified C_q_ cut-off values that yielded maximum Youden indices (Table 2). Reliability of positive qPCR results was assessed with Bland-Altman plots (Figures 3C,D,G,H and Supplementary Table 3) and by calculating the intraclass correlation coefficient between *piaB* and *lytA*. Samples with C_q_ values below the ROC-derived C_q_ criterium (^ROCd^C_q_) demonstrated excellent agreement between *piaB* and *lytA* while samples with a C_q_ value above the ^ROCd^C_q_ threshold displayed poor agreement (Supplementary Table 3). Using the ^ROCd^C_q_ criterium we classified 33% (512/1,549) of minimally processed nasopharyngeal samples as positive for pneumococcus (Figure 2A). It included 52% (489/946) samples from children and 4% (23/603) from adults. For culture-enriched samples application of ^ROCd^C_q_ resulted in 30% (469/1,549) classified as positive including 47% (447/946) samples from children and 4% (22/603) from adults (Figure 2A). With ^ROCd^C_q_, the adults in the Netherlands were again significantly more often positive for pneumococcus in oropharyngeal compared to nasopharyngeal samples whenever tested minimally processed (19% or 59/319 vs. 5% or 15/319; *p* < 0.0001) or culture-enriched (12% or 39/319 vs. 5% or 15/319; *p* < 0.01) (Figure 2B). For culture-enriched nasopharyngeal and oropharyngeal samples the use of a “two-to-tango” approach and ^ROCd^C_q_ resulted in increased positive predictive value (PPV), specificity and agreement (Cohen’s kappa) of qPCR-based detection to culture plus re-culture when compared to use of either *piaB* or *lytA* alone (Supplementary Table 4).

**TABLE 2:** Optimal qPCR cycle threshold C_q_ and corresponding parameters for *Streptococcus pneumoniae* carriage detection in nasopharyngeal (*n* = 1,549) and oropharyngeal (*n* = 319) samples.

Parameter	Sample	Reference	Optimal threshold *piaB* (95% CI)	Youden index *piaB*	Sensitivity *piaB*	Specificity *piaB*	Optimal threshold *lytA* (95% CI)	Youden index *lytA*	Sensitivity *lytA*	Specificity *lytA*
Minimally processed	NP	Primary cultures	37.13 (36.49–37.76)	0.85	0.95	0.90	37.47 (36.44–38.39)	0.82	0.94	0.87
	NP	Primary culture and reculture	37.21 (36.51–38.46)	0.85	0.95	0.90	37.29 (36.52–38.61)	0.83	0.94	0.89
	OP	Primary culture and reculture	38.73 (37.29–39.39)	0.69	0.91	0.78	37.47 (36.52–37.66)	0.69	0.87	0.82
Culture-enriched	NP	Primary cultures	28.71 (26.59–29.69)	0.94	0.98	0.97	29.19 (27.41–30.25)	0.93	0.98	0.95
	NP	Primary culture and reculture	30.26 (29.91–30.47)	0.96	0.98	0.98	29.99 (28.91–30.47)	0.94	0.98	0.96
	OP	Primary culture and reculture	29.78 (28.84–29.93)	0.91	0.96	0.95	30.73 (29.42–30.81)	0.85	0.91	0.94

*Results from qPCR were validated in a receiver operating characteristic curve analysis with culture as reference. With S. pneumoniae isolated from only three samples there was insufficient statistical power to perform the receiver operating characteristic curve analysis on oropharyngeal samples with primary cultures as a reference.*

### Comparison of Molecular Methods to Culturing Live *Streptococcus pneumoniae*

We compared the diagnostic accuracy of detection methods using the combined results of primary diagnostic and qPCR-guided culturing as reference and applying ^ROCd^C_q_ criteria for positivity in qPCRs (Figure 2A). For nasopharyngeal samples alone (Table 3), molecular detection of pneumococcus displayed near-perfect, and substantial agreement to the reference for culture-enriched and minimally processed samples, respectively. This represented significantly reduced agreement for minimally processed compared with culture-enriched samples (*p* < 0.0001). Molecular detection in minimally processed samples identified significantly more samples positive for pneumococcus compared with culture-enriched samples from children (*p* < 0.0001) but not adults (*p* = 1), the difference we attributed to low number of positive nasopharyngeal samples among collected from adults. Exclusively for children, molecular detection of pneumococcus in culture-enriched samples demonstrated significantly increased sensitivity (*p* < 0.001) and specificity (*p* < 0.0001) compared with molecular detection in minimally processed samples.

**TABLE 3:** The accuracy of *Streptococcus pneumoniae* detection in *n* = 1,549 nasopharyngeal samples from children (*n* = 946) and adults (*n* = 603) tested using conventional diagnostic culture and molecular methods applied to DNA extracted from minimally processed and culture-enriched samples and applying ^ROCd^C_q_ thresholds for a sample positivity in qPCRs.

Method	Study population	Percent (n) of positive samples (95%CI)	PPV % (95%CI)	NPV % (95%CI)	Sensitivity % (95%CI)	Specificity % (95%CI)	Concordance % (95%CI)	κ (95%CI)
Primary culture	All	28.7 (445) (26.5–31.0)	100 (99.1–100)	98.6 (97.8–99.2)	96.7 (94.7–98.0)	100 (99.6–100)	99.0 (98.4–99.4)	0.98 (0.96–0.99)
	Children	44.6 (422) (41.5–47.8)	100 (99.1–100)	97.1 (95.3–98.3)	96.6 (94.4–97.9)	100 (99.3–100)	98.4 (97.4–99.0)	0.97 (0.95–0.98)
	Adults	3.8 (23) (2.6–5.7)	100 (85.7–100)	100 (99.3–100)	100 (85.7–100)	100 (99.3–100)	100 (99.4–100)	1
qPCRs on minimally processed samples	All	33.1 (512) (30.8–35.4)	83.2 (79.7–86.2)	96.7 (95.5–97.6)	92.6 (89.8–94.7)	92.1 (90.3–93.6)	92.3 (90.8–93.5)	0.82 (0.79–0.85)
	Children	51.7 (489) (48.5–54.9)	83.6 (80.1–86.7)	93.9 (91.3–95.7)	93.6 (90.9–95.5)	84.3 (80.9–87.2)	88.6 (86.4–90.5)	0.77 (0.73–0.81)
	Adults	3.8 (23) (2.6–5.7)	73.9 (53.5–87.5)	99.0 (97.8–99.5)	73.9 (53.5–87.5)	99.0 (97.8–99.5)	98.0 (96.6–98.9)	0.73 (0.58–0.88)
qPCRs on culture-enriched samples	All	30.3 (469) (28.0–32.6)	95.7 (93.5–97.2)	99.0 (98.2–99.4)	97.6 (95.8–98.7)	98.2 (97.2–98.8)	98.0 (97.2–98.6)	0.95 (0.94–0.97)
	Children	47.3 (447) (44.1–50.4)	96.0 (93.7–97.4)	98.4 (96.9–99.2)	98.2 (96.4–99.1)	96.5 (94.5–97.8)	97.3 (96.0–98.1)	0.94 (0.92–0.97)
	Adults	3.7 (22) (2.4–5.5)	90.9 (72.2–97.5)	99.5 (98.5–99.8)	87.0 (67.9–95.5)	99.7 (98.8–99.9)	99.2 (98.1–99.6)	0.88 (0.78–0.99)

*Measures of diagnostic accuracy were calculated by comparing the number of detected samples positive per method with the number of (n = 460 for all individuals, and n = 437 and n = 23 for nasopharyngeal samples from children and adults, respectively) individuals positive for S. pneumoniae based on isolation of live pneumococcus either from the primary diagnostic or qPCR-guided culture. PPV, positive predictive value; NPV, negative predictive value; 95%CI, 95% confidence interval; κ, Cohen’s kappa where ≤ 0, 0.01–0.20, 0.21–0.40, 0.41–0.60, 0.61–0.80, > 0.81 are interpreted as poor agreement, slight, fair, moderate, substantial, and almost perfect agreement, respectively.*

For paired nasopharyngeal and oropharyngeal samples from adults, the isolation of *S. pneumoniae* in primary or qPCR-guided culture in either nasopharyngeal or oropharyngeal swab was used as the reference in method accuracy analysis (Table 4). Although the sensitivity of *S. pneumoniae* detection with primary nasopharyngeal culture was significantly higher compared with corresponding values for primary oropharyngeal cultures (*p* < 0.001), every detection method when applied to a single sample type displayed only slight to moderate agreement to the reference. Moreover, the sensitivity of *S. pneumoniae* detection with either nasopharyngeal and oropharyngeal primary cultures was significantly lower compared with molecular detection in culture-enriched oropharyngeal samples (*p* < 0.05) and also when compared with molecular detection with either culture-enriched sample of either type (*p* < 0.0001). To this extent, molecular detection in culture-enriched oropharyngeal plus nasopharyngeal samples displayed the highest agreement to the reference out of all other evaluated approaches (Table 4).

**TABLE 4:** The accuracy of *Streptococcus pneumoniae* detection in paired nasopharyngeal and oropharyngeal samples from *n* = 319 adults tested using molecular methods applied to DNA extracted from minimally processed and culture-enriched samples and applying ^ROCd^C_q_ thresholds for a sample positivity in qPCRs.

Method	Positivity in	Percent (n) of positive samples (95%CI)	PPV % (95%CI)	NPV % (95%CI)	Sensitivity % (95%CI)	Specificity % (95%CI)	Concordance % (95%CI)	κ (95%CI)
Primary culture alone	NP	4.7 (15) (2.9–7.6)	100 (79.6–100)	92.8 (89.3–95.2)	40.5 (26.3–56.5)	100 (98.7–100)	93.1 (89.8–95.4)	0.55 (0.36–0.73)
	OP	0.9 (3) (0.3–0.7)	100 (43.9–100)	89.2 (85.3–92.2)	8.1 (2.8–21.3)	100 (98.7–100)	89.3 (85.5–92.3)	0.13 (−0.14–0.41)
	Either NP or OP	5.6 (18) (3.6–8.7)	100 (82.4–100)	93.7 (90.4–95.9)	48.6 (33.4–64.1)	100 (98.7–100)	94.0 (90.9–96.2)	0.63 (0.46–0.79)
qPCRs on minimally processed samples	NP	4.7 (15) (2.9–7.6)	73.3 (48.0–89.1)	91.4 (87.8–94.1)	29.7 (17.5–45.8)	98.6 (96.4–99.4)	90.6 (86.9–93.3)	0.38 (0.17–0.59)
	OP	15.7 (50) (12.1–20.1)	34.0 (22.4–47.8)	92.6 (88.8–95.1)	45.9 (31.0–61.6)	88.3 (84.0–91.5)	83.4 (78.9–87.1)	0.30 (0.12–0.47)
	Either NP or OP	19.1 (61) (15.2–23.8)	41.0 (29.5–53.5)	95.3 (92.0–97.3)	67.6 (51.5–80.4)	87.2 (82.8–90.6)	85.0 (80.6–88.5)	0.43 (0.28–0.58)
qPCRs on culture-enriched samples	NP	4.7 (15) (2.9–7.6)	86.7 (62.1–96.3)	92.1 (88.5–94.6)	35.1 (21.8–51.2)	99.3 (88.3–94.4)	91.8 (88.3–94.4)	0.46 (0.27–0.66)
	OP	12.2 (39) (9.1–16.3)	61.5 (45.9–75.1)	95.4 (92.2–97.3)	64.9 (48.8–78.2)	94.7 (91.4–96.8)	91.2 (87.6–93.9)	0.58 (0.43–0.73)
	Either NP or OP	16.3 (52) (12.7–20.8)	67.5 (53.8–78.5)	99.3 (97.3–99.8)	94.6 (82.3–98.5)	94.0 (90.6–96.2)	94.0 (90.9–96.2)	0.75 (0.65–0.86)

*Measures of diagnostic accuracy were calculated by comparing the number of detected samples positive per method with the overall number of n = 37 individuals identified as carriers of S. pneumoniae based on isolation of live pneumococcus either at the primary diagnostic or qPCR-guided culture and either from nasopharyngeal or oropharyngeal sample. NP, nasopharyngeal; OP, oropharyngeal; PPV, positive predictive value; NPV, negative predictive value; 95%CI, 95% confidence interval; κ, Cohen’s kappa where ≤ 0, 0.01–0.20, 0.21–0.40, 0.41–0.60, 0.61–0.80, > 0.81 are interpreted as poor agreement, slight, fair, moderate, substantial, and almost perfect agreement, respectively.*

### Comparison of Serotype Carriage Detection Methods

With conventional culture (without qPCR-guided additional culture), 29% (445/1,549) of nasopharyngeal samples including 45% (422/946) from children and 4% (23/603) from adults were positive for a serotype (non-typeable pneumococci excluded), as already described above. Next, we assessed the accuracy of molecular methods when applied to detect carriage of pneumococcal serotypes. Supplementary Figure 1 depicts results of serotype detection in culture-enriched samples tested with serotype-specific qPCR assays. None of the samples generated any signal in qPCRs targeting serotype 1, and 23F and serogroup 18, nor was positive for any of these serotypes by culture. The assays targeting serotypes 4, 5 showed a lack of specificity and the assay targeting serotype 17F showed lack of sensitivity when applied to both, nasopharyngeal and to oropharyngeal samples. Results of these three assays were excluded from analysis.

**FIGURE 4:**
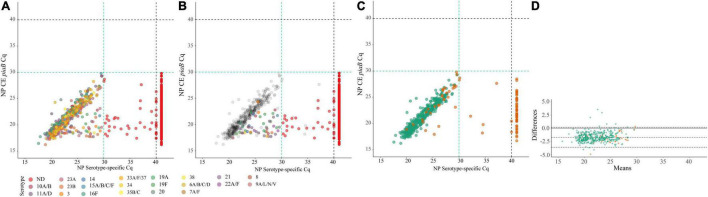
Scatter plots **(A–C)** displaying correlation between cycle threshold (C_q_) from real-time PCR (qPCR) assays targeting the *Streptococcus pneumoniae piaB* and serotype/serogroup specific signal detected with qPCR for nasopharyngeal samples classified positive for *S. pneumoniae* according to ^ROCd^C_q_ criterium (green dashed lines). Bland-Altman plot **(D)** displaying agreement between *piaB* and dominant serotype detected by qPCR. Each dot represents an individual serotype carriage event detected by qPCR in culture-enriched nasopharyngeal sample **(A–C)**. Dots in **(A)** depicts serotypes detected with qPCR. Dots are color-coded according to serotypes/serogroup targeted in an assay (see legend). Color dots in **(B)** depict subdominant serotypes detected qPCR while gray dots mark dominant serotypes. Red dots in **(A,B)** depict samples not generating any signal in serotype/serogroup-specific qPCRs or with the signal of C_q_ higher than the ^ROCd^C_q_ threshold, hence classified as negative for a serotype. In **(C,D)** green dots mark samples with congruent serotype between culture and molecular methods and orange dots mark samples with non-congruent result. In **(D)** shaded gray areas mark the 95% confidence interval of the upper limit of agreement, bias and lower limit of agreement. The continuous line marks the line of equality.

**FIGURE 5:**
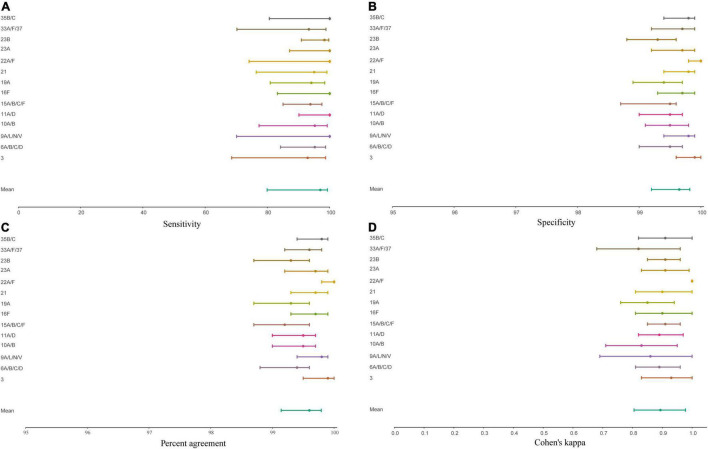
Forest plot displaying the point estimate and 95% confidence of intervals sensitivity **(A)**, specificity **(B)**, concordance **(C)**, and Cohen’s kappa **(D)** for molecular diagnostic tests applied to culture-enriched samples when compared to isolation of *S. pneumoniae* strains of particular serotype/serogroup from nasopharyngeal swabs collected in the study. Graphs displayed results for serotypes/serogroups that have been cultured from > 5 nasopharyngeal samples.

Four-hundred and twenty-three nasopharyngeal samples were positive for one or more serotypes targeted in qPCRs either by culture (*n* = 393 samples) or with molecular methods (*n* = 411 samples) and applying ^ROCd^C_q_ criterium for positivity for a serotype. It included 42% (395/946) samples from children and 3% (16/603) from adults. Altogether, there were *n* = 479 serotypes carriage events detected by testing nasopharyngeal samples with either conventional culture or molecular methods (Supplementary Table 5). For serotypes targeted by qPCRs detected in nasopharyngeal samples the results of molecular detection displayed excellent agreement (ICC 0.93, 95% CI 0.92–0.94) with *piaB* and *lytA* C_q_s (Figure 4) and almost perfect agreement (Cohen’s kappa > 0.81) with isolation of live strain of a particular serotype from nasopharyngeal swab (Figure 5). Also, results of serotype detection in oropharyngeal samples from adults displayed excellent agreement with *piaB* and *lytA* C_q_s (ICC 0.96, 95% CI 0.92–0.98) (Supplementary Figure 2). Finally, there was near-perfect agreement between overall serotypes carriage events detected by qPCR compared with detected by culture (Table 5). Multiple-serotype carriage events were significantly more often detected using molecular methods compared with culture (*p* < 0.0001). Furthermore, despite a limited number of different serotypes tested by qPCR, observed multiple-serotype carriage frequencies were not significantly different from expected frequencies based on molecular detection on culture-enriched NP samples (11% vs. 9%, respectively; one proportion *Z*-test, *p* = 0.2669) unlike detection of multiple-serotype carriage by culture which significantly underestimated expected frequencies (2% vs. 9%, respectively; one-proportion *Z*-test, *p* < 0.0001).

**TABLE 5:** The accuracy of *Streptococcus pneumoniae* serotype detection in *n* = 1,549 nasopharyngeal samples from children (*n* = 946) and adults (*n* = 603) tested using molecular methods applied to DNA extracted culture-enriched samples.

Study population	PPV % (95%CI)	NPV % (95%CI)	Sensitivity % (95%CI)	Specificity % (95%CI)	Concordance % (95%CI)	κ (95%CI)
All (*n* = 1,549)	100 (99.0–100)	98.7 (97.9–99.2)	96.2 (93.8–97.7)	100 (99.7–100)	99.0 (99.7–100)	0.97 (0.96–0.99)
Children (*n* = 946)	98.1 (96.6–98.9)	98.1 (96.6–98.9)	97.1 (94.8–98.4)	100 (99.3–100)	98.8 (97.9–99.3)	0.98 (0.96–0.99)
Adults (*n* = 603)	100 (78.5–100)	99.3 (98.3–99.7)	77.8 (54.8–91.0)	100 (99.3–100)	99.3 (98.3–99.7)	0.87 (0.75–1)

*Measures of diagnostic accuracy were calculated by comparing the numbers of serotype carriage events detected with molecular methods with n = 393 samples from which serotypes were cultured that were targeted by serotype-specific qPCR assays. PPV, positive predictive value; NPV, negative predictive value; 95%CI, 95% confidence interval; κ, Cohen’s Kappa where ≤ 0, 0.01–0.20, 0.21–0.40, 0.41–0.60, 0.61–0.80, > 0.81 are interpreted as poor agreement, slight, fair, moderate, substantial, and almost perfect agreement, respectively.*

### Interlaboratory Reproducibility of Molecular Methods

To evaluate the reproducibility of molecular methods and to assess the agreement in laboratory results between the Netherlands and England we processed a subset of culture-enriched samples from both countries in both laboratories. Results for *piaB* and *lytA* qPCRs demonstrated good reliability between both laboratories (Supplementary Table 6). We observed near-perfect agreement identifying culture-enriched samples as positive for pneumococcus with molecular methods, and substantial agreement for minimally processed nasopharyngeal samples (Supplementary Table 7). For culture-enriched nasopharyngeal samples we evaluated agreement between both laboratories for serotype carriage detection by qPCR. Overall, near-perfect agreement was observed (Cohen’s kappa 0.82, 95% CI 0.74–0.90).

## Discussion

In the current study we have demonstrated that molecular methods exhibit near-perfect agreement to conventional culture in the detection and serotyping of *S. pneumoniae* in children and adults when a nasopharyngeal swab is the only sample tested. Furthermore, we have observed increased sensitivity of *S. pneumoniae* carriage detection among adults by testing oropharyngeal samples with molecular methods and conducting qPCR-guided culturing. We highlight several statistical procedures that can be used to evaluate the reliability of molecular results and enhance the specificity of molecular methods for the detection of live *S. pneumoniae*.

The current gold standard method for carriage detection is the isolation of live pneumococci from cultures of deep trans-nasal nasopharyngeal swab, in adults complemented with culture of a swab collected trans-orally (OP) (Satzke et al., 2013). However, the gold standard lacks sensitivity in case of low-density carriage or when applied to poly-microbial samples in which *S. pneumoniae* is not a dominant bacterium and it does not allow the detection of co-carriage of pneumococcal strains (Heffron, 1939; Trzcinski et al., 2013; Wyllie et al., 2014, 2016a; Almeida et al., 2020). Since the density of pneumococcal carriage episodes in adults is much lower than in children (Trzcinski et al., 2013), sampling multiple sites increases sensitivity of carriage detection (Trzcinski et al., 2013; Krone et al., 2014; Wyllie et al., 2016a). However, carriage surveillance based exclusively on primary diagnostic cultures of nasopharyngeal and oropharyngeal samples often provides low quality data in adults. This limitation of the gold standard method is of particular concern for surveillance of carriage in older adults (Almeida et al., 2020; Miellet et al., 2020), the age group with the largest incidence and burden of pneumococcal pneumonia and invasive pneumococcal disease (Jansen et al., 2009; Welte et al., 2012) as carriage is often reported to be virtually absent when the gold standard method is the only applied (Krone et al., 2014; Arguedas et al., 2020).

To overcome these limitations, we complement conventional culture with molecular methods to improve the overall sensitivity of *S. pneumoniae* carriage detection (Trzcinski et al., 2013; Wyllie et al., 2014, 2016a,^2016^; Krone et al., 2015; Miellet et al., 2020). This approach is particularly effective when applied to highly poly-microbial samples from the oral niche. To validate this method, we have compared the performance of molecular methods to the gold standard. While demonstrating near-perfect agreement with primary nasopharyngeal cultures from children, application of qPCR-based methods still significantly increased the number of carriers detected. Near perfect agreement was also observed in adults when analysis focused explicitly on nasopharyngeal swabs. In adults, application of qPCR-based methods to nasopharyngeal samples did not increase sensitivity of carriage detection.

Importantly, in adults testing oropharyngeal samples changed the results dramatically. By revisiting samples negative in primary culture yet positive by qPCR with qPCR-guided culturing we have significantly increased the number of adult carriers from whom viable pneumococci were isolated. It demonstrates that molecular methods can improve the overall sensitivity of *S. pneumoniae* detection, a result in line with previous reports by us and others (Trzcinski et al., 2013; Krone et al., 2014, 2015; Wyllie et al., 2016a; Almeida et al., 2021). It also highlights that the gold standard method applied to oropharyngeal samples severely underestimates presence of *S. pneumoniae* in adults as qPCR-guided culturing increased by 7.7-fold the number of oropharyngeal samples from which viable pneumococci were isolated. Interestingly, there is evidence that testing oropharyngeal in addition to nasopharyngeal samples substantially enhances sensitivity of carriage detection also in children (Korona-Glowniak et al., 2011).

Concerns have been raised that the molecular methods are overly sensitive and lack specificity for the detection of live bacteria as “relic DNA” (DNA from non-intact cells) could be detected as well (Lennon et al., 2018). Indeed, live pathogens are less likely to be cultured from samples displaying weak positivity by qPCR (Wyllie et al., 2014; Miellet et al., 2021). While this may reflect limitations in current culturing techniques, it could also indicate presence of relic DNA that may reflect recent exposure to pneumococcus (e.g., prior to antibiotic therapy) instead of colonization proper (Lennon et al., 2018). Alternatively, suboptimal sample transport and storage conditions can also greatly limit the success rate of culturing in a carriage study. The addition of the culture-enrichment procedure prior to molecular detection reduces the risk of misclassifying these events as carriage, thus improving the specificity of detection for live pneumococci. Culture-enrichment also increases the sensitivity of carriage surveillance, in particular for poly-microbial samples (Trzcinski et al., 2013; Wyllie et al., 2014, 2016a,b; Krone et al., 2015; Rodrigues et al., 2019; Miellet et al., 2020, 2021; Almeida et al., 2021). Indeed, we observed that *S. pneumoniae* detection in culture-enriched samples displayed significantly increased, sensitivity, specificity, and agreement with culture and qPCR-guided culture when compared with detection in minimally processed samples.

To further improve the specificity of molecular methods for live pneumococci we performed ROC curve analysis to estimate with the Youden index C_q_ cut-off values that display an optimal combination of sensitivity and specificity for samples positive for *S. pneumoniae* by culture in primary cultures or qPCR-guided cultures (Nutz et al., 2011). This approach excluded samples that exhibited minimal positivity for targeted genes by qPCR or displayed poor agreement between *piaB* and *lytA* genes as shown in Bland-Altman graphs and with the intra-class correlation coefficient. The use of the Youden index in a ROC curve analysis to identify optimal C_q_ cut-off values enhances the specificity of molecular methods for detection of live pneumococcus, and the ^ROCd^C_q_ criteria can be used to direct qPCR-guided culturing. The success of this approach is dependent on culturing methods, therefore care should be taken to employ sensitive culturing techniques, such as the use of selective culture plates (Satzke et al., 2013). Furthermore, for molecular methods to be informative for culturing efforts DNA extraction should be performed irrespective of the identification of pneumococcal growth in cultures. ROC curve analysis with the Youden index may yield overly stringent C_q_ cut-off values if qPCR-guided culturing efforts are not conducted, which could result in classifying samples containing live pneumococci as negative by qPCR. Recent advances in culturing techniques may further enhance the specificity of molecular methods for the detection of live *S. pneumoniae* (York et al., 2021).

Some studies have cautioned against the use of molecular methods due to the presence of pneumococcal genes among commensal streptococci (Carvalho Mda et al., 2012, 2013; Boelsen et al., 2020; Ganaie et al., 2021). This phenomenon is likely to be common among bacterial species co-existing in a shared niche (Kroll et al., 1998; Kilian et al., 2014; Price et al., 2015) and of particular concern for highly poly-microbial samples, hence careful selection of targeted genes is important (Greve and Moller, 2012). As previously described by our group (Trzcinski et al., 2013; Krone et al., 2015; Wyllie et al., 2016b) and others (Tavares et al., 2019) a “two-to-tango” approach, quantifying both *piaB* and *lytA* genes with molecular methods enhances the specificity of *S. pneumoniae* detection in poly-microbial samples than either target alone. This approach allows for measurements to be evaluated in a reliability analysis on a per sample basis (Bland and Altman, 1986) and for bias between measurements to be identified. While systemic biases between quantified genes could reflect dissimilarities between qPCR assay efficiencies, non-systemic biases can arise due to the presence of a gene or closely related sequence in DNA from other bacterial species. In case of multiple serotype carriage, serotype-specific abundance of the dominant serotype should display high agreement to targeted genes used to detect pneumococcus (e.g., *piaB* and *lytA*) while serotype-specific abundances of non-dominant serotypes may display reduced agreement. Serotypes that exhibit greater abundances than *piaB* or *lytA*, or appear to be present in samples classified as negative for *S. pneumoniae* are likely to be due to commensal streptococci harboring genes involved in the biosynthesis of the pneumococcal polysaccharide capsule (Lessa et al., 2018; Pimenta et al., 2019). As such, certain serotype-specific assays may be non-reliable in poly-microbial sample types. Importantly, this also concerns serotype-specific assays targeting vaccine, such as serotypes 4 and 5 (Carvalho Mda et al., 2012, 2013; Wyllie et al., 2014, 2016a,^2017^; Lessa et al., 2018; Pimenta et al., 2019; Ganaie et al., 2021).

Insights into the co-occurrence of multiple serotypes in carriage is critical for understanding the dynamics of the serotypes during colonization, host-to-host transmission, and carriage progression into disease. Detection of secondary strains present in co-carriage is also important when distinguishing between unmasking and serotype replacement in assessment of pneumococcal vaccines impact (Huebner et al., 2000). However, as demonstrated in our study, and described previously the gold standard method does not readily allow detection of multiple serotypes, an event that is likely to occur often in carriage (Huebner et al., 2000). Furthermore, using our methodology we have observed no significant difference in observed and expected frequencies of multiple-serotype carriage despite what has been reported previously by others who exclusively used data from conventional culture as method of detection (Numminen et al., 2013).

An important strength of our study is the application of the procedure to two different carriage studies conducted in two different countries, and the evaluation of reproducibility in an interlaboratory comparison. Another strength of our study is that the procedure described is flexible and can be readily adapted to the carriage surveillance of other bacterial pathogens, such as *Neisseria meningitidis* (Miellet et al., 2021), for which detection of live bacteria is important or for which conventional culture may display insufficient sensitivity.

Our study had a number of limitations, the impact of testing oropharyngeal samples was only evaluated for Dutch adults and not adults sampled in England nor for any children. Since oropharyngeal samples tested were collected explicitly from Dutch adults, our findings could be unique for that demographic group and geographic location. In the protocol described we only used *piaB* and *lytA* to detect *S. pneumoniae* with molecular methods and we did not evaluate the procedure with alternative targets described by others (Shafeeq et al., 2013; Tavares et al., 2019). Furthermore, we tested for a limited number of serotypes by qPCR. Finally, not all serotypes were shown to be equally reliably detected with molecular methods and for a number of qPCR assays we were not able to identify the serotype within a serogroup.

In summary, we argue that accurate detection of pneumococcal carriage using qPCR requires concordant quantification of two genes (“two-targets-to-tango”) to classify a sample as positive for pneumococcus. Similarly, qPCR-based detection requires concordance between pneumococcal and serotype-specific quantification to assure specificity of the method (“two-targets-to-tango”). We provide evidence that accurate detection of pneumococcal carriage in adults requires at least testing of both, nasopharyngeal and oropharyngeal samples (“two-samples-to-tango”) and requires molecular detection to be intertwined with culture (“two methods-to-tango”). Finally, we advise revisiting samples for qPCR-guided culturing (“two-cultures-to-tango”) when positive by qPCR but negative at primary diagnostic culture. The use of qPCR-guided culturing is of utmost importance for oropharyngeal swabs.

We have outlined the procedure that enhances the specificity of molecular methods for the surveillance of pneumococci and of pneumococcal serotypes in nasopharyngeal and oropharyngeal samples. Our results demonstrate near-perfect agreement between conventional culture and molecular methods when applied to nasopharyngeal samples from children. We have shown that the sensitivity of *S. pneumoniae* carriage surveillance can be greatly enhanced by complementing conventional culture with qPCRs. In adults, testing oropharyngeal on the top of nasopharyngeal samples was of paramount importance for accuracy of pneumococcal carriage detection. Studies investigating impact of testing oropharyngeal samples on detection of pneumococcal carriage in children are needed.

## Data Availability Statement

The raw data supporting the conclusions of this article will be made available by the authors, without undue reservation.

## Ethics Statement

The studies involving human participants were reviewed and approved by the Medical Ethics Committee Noord Holland (NTR5405 on http://www.trialregister.nl) and by the NHS Health Research Authority and the London Fulham Research Ethics Committee (reference 15/LO/0458; on clinicaltrials.gov reference NCT02522546). Written informed consent to participate in this study was provided by the participants’ legal guardian/next of kin.

## Author Contributions

ES and KT had an idea and initiated the study. NR, EM, NF, ES, and KT secured financial support. NF and KT led the project. AW-M, PB, CS, MH, NR, EM, and NF conducted carriage studies, collected the data, and provided study materials. WM, JV, and KT developed, validated laboratory methods, and wrote the laboratory protocol. WM, JV, DL, PB, TN, SM, RT, SE, and CS analyzed samples and collected the data. WM, RM, and KT contributed analytical tools. WM, JV, and DL curated the data. WM, DL, NF, and KT managed the study. WM and KT performed formal analysis of study data, visualized presentation of the results, and drafted the manuscript. All authors amended, critically reviewed, and commented on the final manuscript.

## Conflict of Interest

Public Health England (PHE) has provided vaccine manufacturers (GSK, Pfizer, Sanofi) with post-marketing surveillance reports on pneumococcal infection which the companies are required to submit to the UK Licensing authority in compliance with their Risk Management Strategy. A cost recovery charge is made for these reports. PHE’s Respiratory and Vaccine Preventable Bacteria Reference Unit has received unrestricted research grants from Pfizer to participate in pneumococcal surveillance projects. KT received funds for an unrestricted research grant from GlaxoSmithKline Biologicals SA, consultation fees, fees for participation in advisory boards, speaking fees and funds for unrestricted research grants from Pfizer, and fees for participating in advisory boards from Merck Sharp and Dohme, all paid directly to his home institution. Except for the funds from GlaxoSmithKline Biologicals SA none was received in the relation to the work reported here. The remaining authors declare that the research was conducted in the absence of any commercial or financial relationships that could be construed as a potential conflict of interest.

## Publisher’s Note

All claims expressed in this article are solely those of the authors and do not necessarily represent those of their affiliated organizations, or those of the publisher, the editors and the reviewers. Any product that may be evaluated in this article, or claim that may be made by its manufacturer, is not guaranteed or endorsed by the publisher.

## Footnotes

1
http://www.trialregister.nl

2
https://github.com/wmiellet/test-comparison-R

## Supplementary Material

Supplementary Figure 1 Results of molecular method-based quantification of serogroups/serotypes-specific sequences in DNA from culture-enriched nasopharyngeal and oropharyngeal samples. Individual scatter plots depict results of a single serotype-specific or serogroup-specific qPCR assay as labeled above the panel. Each symbol represents an individual sample: pale-red dots represent nasopharyngeal samples from children; blue triangles and green triangles represent nasopharyngeal and oropharyngeal samples from adults, respectively. Sample was classified as positive for a serotype/serogroup with molecular method when the signal detected by qPCR for *piaB* (*X*-axis) and serotype/serogroup (*Y*-axis) were both below the ^ROCd^C*_*q*_* cut-off threshold of 30 C*_*q*_*. Only samples that generated a signal of C*_*q*_* < 40 in a particular serogroup/serotype-specific qPCR are depicted. Symbols of C*_*q*_* > 40 for *piaB* depict individual samples or pools of samples negative for pneumococcus by qPCR. In the panel depicting qPCR results for serogroup 6 all symbols represent samples identified as positive for serotypes 6C or 6D except for red circles depicting two samples identified as positive for serotype 6A or 6B, both positive for serotype 6A by culture. In the panel depicting qPCR results for serogroup 22 all symbols represent positivity for serotype 22F. None of the samples generated any signal in qPCRs targeting serotype 1, serotype 23F and serogroup 18. In addition, no samples were identified as positive for serogroup 12 according to study criteria.

Supplementary Figure 2 Scatter plot displaying correlation between cycle threshold (C_q_) from real-time PCR (qPCR) assays targeting the *Streptococcus pneumoniae piaB* and serotype/serogroup specific signal detected with qPCR for culture-enriched (CE) oropharyngeal samples from adults classified positive for *S. pneumoniae* according to ^ROCd^C_q_ criterium (green dashed lines). Dots depicts serotypes detected with qPCR. Dots are color-coded according to serotypes/serogroup targeted in an assay (see legend).

## References

[B1] Almeida S. T. Paulo A. C. Froes F. de Lencastre H. Sa-Leao R. (2021). Dynamics of pneumococcal carriage in adults: a new look at an old paradigm. *J. Infect. Dis.* 223 1590–1600. 10.1093/infdis/jiaa558 32877517

[B2] Almeida S. T. Pedro T. Paulo A. C. de Lencastre H. Sa-Leao R. (2020). Re-evaluation of *Streptococcus pneumoniae* carriage in Portuguese elderly by qPCR increases carriage estimates and unveils an expanded pool of serotypes. *Sci. Rep.* 10:8373. 10.1038/s41598-020-65399-x 32433504 PMC7239868

[B3] Altman D. G. Bland J. M. (2003). Interaction revisited: the difference between two estimates. *BMJ* 326:219. 10.1136/bmj.326.7382.219 12543843 PMC1125071

[B4] Arguedas A. Trzcinski K. O’Brien K. L. Ferreira D. M. Wyllie A. L. Weinberger D. (2020). Upper respiratory tract colonization with *Streptococcus pneumoniae* in adults. *Expert Rev. Vaccines* 19 353–366. 10.1080/14760584.2020.1750378 32237926

[B5] Auranen K. Rinta-Kokko H. Goldblatt D. Nohynek H. O’Brien K. L. Satzke C. (2013). Colonisation endpoints in *Streptococcus pneumoniae* vaccine trials. *Vaccine* 32 153–158. 10.1016/j.vaccine.2013.08.061 24016803

[B6] Azzari C. Moriondo M. Cortimiglia M. Valleriani C. Canessa C. Indolfi G. (2012). Potential serotype coverage of three pneumococcal conjugate vaccines against invasive pneumococcal infection in Italian children. *Vaccine* 30 2701–2705. 10.1016/j.vaccine.2011.12.008 22178097

[B7] Azzari C. Moriondo M. Indolfi G. Cortimiglia M. Canessa C. Becciolini L. (2010). Realtime PCR is more sensitive than multiplex PCR for diagnosis and serotyping in children with culture negative pneumococcal invasive disease. *PLoS One* 5:e9282. 10.1371/journal.pone.0009282 20174571 PMC2824814

[B8] Berical A. C. Harris D. Dela Cruz C. S. Possick J. D. (2016). Pneumococcal vaccination strategies. An update and perspective. *Ann. Am. Thorac. Soc.* 13 933–944. 10.1513/AnnalsATS.201511-778FR 27088424 PMC5461988

[B9] Bland J. M. Altman D. G. (1986). Statistical methods for assessing agreement between two methods of clinical measurement. *Lancet* 1 307–310. 10.1016/s0140-6736(86)90837-8 2868172

[B10] Boelsen L. K. Dunne E. M. Gould K. A. Ratu F. T. Vidal J. E. Russell F. M. (2020). The challenges of using oropharyngeal samples to measure pneumococcal carriage in adults. *mSphere* 5:e00478-20. 10.1128/mSphere.00478-20 32727860 PMC7392543

[B11] Bogaert D. De Groot R. Hermans P. W. (2004). *Streptococcus pneumoniae* colonisation: the key to pneumococcal disease. *Lancet Infect. Dis.* 4 144–154. 10.1016/S1473-3099(04)00938-7 14998500

[B12] Carvalho Mda G. Bigogo G. M. Junghae M. Pimenta F. C. Moura I. Roundtree A. (2012). Potential nonpneumococcal confounding of PCR-based determination of serotype in carriage. *J. Clin. Microbiol.* 50 3146–3147. 10.1128/JCM.01505-12 22760044 PMC3421822

[B13] Carvalho Mda G. Pimenta F. C. Moura I. Roundtree A. Gertz R. E. Jr. Li Z. (2013). Non-pneumococcal mitis-group streptococci confound detection of pneumococcal capsular serotype-specific loci in upper respiratory tract. *PeerJ* 1:e97. 10.7717/peerj.97 23825797 PMC3698467

[B14] Carvalho Mda G. Tondella M. L. McCaustland K. Weidlich L. McGee L. Mayer L. W. (2007). Evaluation and improvement of real-time PCR assays targeting lytA, ply, and psaA genes for detection of pneumococcal DNA. *J. Clin. Microbiol.* 45 2460–2466. 10.1128/JCM.02498-06 17537936 PMC1951257

[B15] Dagan R. Muallem M. Melamed R. Leroy O. Yagupsky P. (1997). Reduction of pneumococcal nasopharyngeal carriage in early infancy after immunization with tetravalent pneumococcal vaccines conjugated to either tetanus toxoid or diphtheria toxoid. *Pediatr. Infect. Dis. J.* 16 1060–1064. 10.1097/00006454-199711000-00011 9384340

[B16] Flasche S. Lipsitch M. Ojal J. Pinsent A. (2020). Estimating the contribution of different age strata to vaccine serotype pneumococcal transmission in the pre vaccine era: a modelling study. *BMC Med.* 18:129. 10.1186/s12916-020-01601-1 32517683 PMC7285529

[B17] Flasche S. Van Hoek A. J. Sheasby E. Waight P. Andrews N. Sheppard C. (2011). Effect of pneumococcal conjugate vaccination on serotype-specific carriage and invasive disease in England: a cross-sectional study. *PLoS Med.* 8:e1001017. 10.1371/journal.pmed.1001017 21483718 PMC3071372

[B18] Ganaie F. Branche A. R. Peasley M. Rosch J. W. Nahm M. H. (2021). Oral streptococci expressing pneumococci-like cross-reactive capsule types can affect WHO recommended pneumococcal carriage procedure. *Clin. Infect. Dis.* [Epub ahead of print]. 10.1093/cid/ciab1003 34891152 PMC9464077

[B19] Greve T. Moller J. K. (2012). Accuracy of using the lytA gene to distinguish *Streptococcus pneumoniae* from related species. *J. Med. Microbiol.* 61(Pt 4), 478–482. 10.1099/jmm.0.036574-0 22135022

[B20] Heffron R. (1939). *Pneumonia; with Special Reference to Pneumococcus Lobar Pneumonia.* Oxford: Oxford University Press.

[B21] Huebner R. E. Dagan R. Porath N. Wasas A. D. Klugman K. P. (2000). Lack of utility of serotyping multiple colonies for detection of simultaneous nasopharyngeal carriage of different pneumococcal serotypes. *Pediatr. Infect. Dis. J.* 19 1017–1020. 10.1097/00006454-200010000-00019 11055610

[B22] Jansen A. G. Rodenburg G. D. de Greeff S. C. Hak E. Veenhoven R. H. Spanjaard L. (2009). Invasive pneumococcal disease in the Netherlands: syndromes, outcome and potential vaccine benefits. *Vaccine* 27 2394–2401. 10.1016/j.vaccine.2009.01.127 19428856

[B23] Kapatai G. Sheppard C. L. Al-Shahib A. Litt D. J. Underwood A. P. Harrison T. G. (2016). Whole genome sequencing of Streptococcus pneumoniae: development, evaluation and verification of targets for serogroup and serotype prediction using an automated pipeline. *PeerJ* 4:e2477. 10.7717/peerj.2477 27672516 PMC5028725

[B24] Kilian M. Riley D. R. Jensen A. Bruggemann H. Tettelin H. (2014). Parallel evolution of *Streptococcus pneumoniae* and Streptococcus mitis to pathogenic and mutualistic lifestyles. *mBio* 5:e1490-14. 10.1128/mBio.01490-14 25053789 PMC4120201

[B25] Koo T. K. Li M. Y. A. (2016). Guideline of selecting and reporting intraclass correlation coefficients for reliability research. *J. Chiropr. Med.* 15 155–163. 10.1016/j.jcm.2016.02.012 27330520 PMC4913118

[B26] Korona-Glowniak I. Niedzielski A. Malm A. (2011). Upper respiratory colonization by *Streptococcus pneumoniae* in healthy pre-school children in south-east Poland. *Int. J. Pediatr. Otorhinolaryngol.* 75 1529–1534. 10.1016/j.ijporl.2011.08.021 21940056

[B27] Kroll J. S. Wilks K. E. Farrant J. L. Langford P. R. (1998). Natural genetic exchange between Haemophilus and Neisseria: intergeneric transfer of chromosomal genes between major human pathogens. *Proc. Natl. Acad. Sci. U.S.A.* 95 12381–12385. 10.1073/pnas.95.21.12381 9770495 PMC22840

[B28] Krone C. L. van de Groep K. Trzcinski K. Sanders E. A. Bogaert D. (2014). Immunosenescence and pneumococcal disease: an imbalance in host-pathogen interactions. *Lancet Respir. Med.* 2 141–153. 10.1016/S2213-2600(13)70165-6 24503269

[B29] Krone C. L. Wyllie A. L. van Beek J. Rots N. Y. Oja A. E. Chu M. L. (2015). Carriage of *Streptococcus pneumoniae* in aged adults with influenza-like-illness. *PLoS One* 10:e0119875. 10.1371/journal.pone.0119875 25789854 PMC4366201

[B30] Landis J. R. Koch G. G. (1977). The measurement of observer agreement for categorical data. *Biometrics* 33 159–174. 10.2307/2529310 843571

[B31] Lennon J. T. Muscarella M. E. Placella S. A. Lehmkuhl B. K. (2018). How, When, and Where Relic DNA affects microbial diversity. *mBio* 9:e00637-18. 10.1128/mBio.00637-18 29921664 PMC6016248

[B32] Lessa F. C. Milucky J. Rouphael N. G. Bennett N. M. Talbot H. K. Harrison L. H. (2018). *Streptococcus mitis* expressing pneumococcal serotype 1 Capsule. *Sci. Rep.* 8:17959. 10.1038/s41598-018-35921-3 30568178 PMC6299277

[B33] McHugh M. L. (2012). Interrater reliability: the kappa statistic. *Biochem. Med.* 22 276–282. 10.11613/bm.2012.031 PMC3900052 23092060

[B34] Miellet W. R. Mariman R. Pluister G. de Jong L. J. Grift I. Wijkstra S. (2021). Detection of *Neisseria meningitidis* in saliva and oropharyngeal samples from college students. *Sci. Rep.* 11:23138. 10.1038/s41598-021-02555-x 34848796 PMC8632920

[B35] Miellet W. R. van Veldhuizen J. Nicolaie M. A. Mariman R. Bootsma H. J. Bosch T. (2020). Influenza-like illness exacerbates pneumococcal carriage in older adults. *Clin. Infect. Dis.* 73 e2680–e2689. 10.1093/cid/ciaa1551 33124669

[B36] Numminen E. Cheng L. Gyllenberg M. Corander J. (2013). Estimating the transmission dynamics of *Streptococcus pneumoniae* from strain prevalence data. *Biometrics* 69 748–757. 10.1111/biom.12040 23822205

[B37] Nutz S. Doll K. Karlovsky P. (2011). Determination of the LOQ in real-time PCR by receiver operating characteristic curve analysis: application to qPCR assays for Fusarium verticillioides and F. proliferatum. *Anal. Bioanal. Chem.* 401 717–726. 10.1007/s00216-011-5089-x 21603916 PMC3132422

[B38] O’Brien K. L. Wolfson L. J. Watt J. P. Henkle E. Deloria-Knoll M. McCall N. (2009). Burden of disease caused by *Streptococcus pneumoniae* in children younger than 5 years: global estimates. *Lancet* 374 893–902. 10.1016/S0140-6736(09)61204-6 19748398

[B39] Pimenta F. Gertz R. E. Jr. Park S. H. Kim E. Moura I. Milucky J. (2019). *Streptococcus infantis*, *Streptococcus mitis*, and *Streptococcus oralis* strains with highly similar cps5 loci and antigenic relatedness to serotype 5 Pneumococci. *Front. Microbiol.* 9:3199. 10.3389/fmicb.2018.03199 30671034 PMC6332807

[B40] Pimenta F. C. Roundtree A. Soysal A. Bakir M. du Plessis M. Wolter N. (2013). Sequential triplex real-time PCR assay for detecting 21 pneumococcal capsular serotypes that account for a high global disease burden. *J. Clin. Microbiol.* 51 647–652. 10.1128/JCM.02927-12 23224094 PMC3553924

[B41] Price E. P. Sarovich D. S. Nosworthy E. Beissbarth J. Marsh R. L. Pickering J. (2015). *Haemophilus influenzae*: using comparative genomics to accurately identify a highly recombinogenic human pathogen. *BMC Genomics* 16:641. 10.1186/s12864-015-1857-x 26311542 PMC4551764

[B42] Rodrigues F. Christensen H. Morales-Aza B. Sikora P. Oliver E. Oliver J. (2019). Viable *Neisseria meningitidis* is commonly present in saliva in healthy young adults: non-invasive sampling and enhanced sensitivity of detection in a follow-up carriage study in Portuguese students. *PLoS One* 14:e0209905. 10.1371/journal.pone.0209905 30742640 PMC6370198

[B43] Satzke C. Turner P. Virolainen-Julkunen A. Adrian P. V. Antonio M. Hare K. M. (2013). Standard method for detecting upper respiratory carriage of Streptococcus pneumoniae: updated recommendations from the World Health Organization Pneumococcal Carriage Working Group. *Vaccine* 32 165–179. 10.1016/j.vaccine.2013.08.062 24331112

[B44] Shafeeq S. Kuipers O. P. Kloosterman T. G. (2013). Cellobiose-mediated gene expression in Streptococcus pneumoniae: a repressor function of the novel GntR-type regulator BguR. *PLoS One* 8:e57586. 10.1371/journal.pone.0057586 23469031 PMC3585215

[B45] Sleeman K. L. Griffiths D. Shackley F. Diggle L. Gupta S. Maiden M. C. (2006). Capsular serotype-specific attack rates and duration of carriage of *Streptococcus pneumoniae* in a population of children. *J. Infect. Dis.* 194 682–688. 10.1086/505710 16897668

[B46] Slotved H. C. (2016). Other age groups than children need to be considered as carriers of Streptococcal pneumoniae serotypes. *Hum. Vaccin. Immunother.* 12 2670–2674. 10.1080/21645515.2016.1197451 27322025 PMC5084994

[B47] Southern J. Andrews N. Sandu P. Sheppard C. L. Waight P. A. Fry N. K. (2018). Pneumococcal carriage in children and their household contacts six years after introduction of the 13-valent pneumococcal conjugate vaccine in England. *PLoS One* 13:e0195799. 10.1371/journal.pone.0195799 29799839 PMC5969732

[B48] Tavares D. A. Handem S. Carvalho R. J. Paulo A. C. de Lencastre H. Hinds J. (2019). Identification of *Streptococcus pneumoniae* by a real-time PCR assay targeting SP2020. *Sci. Rep.* 9:3285. 10.1038/s41598-019-39791-1 30824850 PMC6397248

[B49] Trzcinski K. Bogaert D. Wyllie A. Chu M. L. van der Ende A. Bruin J. P. (2013). Superiority of trans-oral over trans-nasal sampling in detecting *Streptococcus pneumoniae* colonization in adults. *PLoS One* 8:e60520. 10.1371/journal.pone.0060520 23555985 PMC3610877

[B50] Turner P. Hinds J. Turner C. Jankhot A. Gould K. Bentley S. D. (2011). Improved detection of nasopharyngeal cocolonization by multiple pneumococcal serotypes by use of latex agglutination or molecular serotyping by microarray. *J. Clin. Microbiol.* 49 1784–1789. 10.1128/JCM.00157-11 21411589 PMC3122683

[B51] Valente C. de Lencastre H. Sa-Leao R. (2013). Selection of distinctive colony morphologies for detection of multiple carriage of Streptococcus pneumoniae. *Pediatr. Infect. Dis. J.* 32 703–704. 10.1097/INF.0b013e31828692be 23838735

[B52] van Gils E. J. Veenhoven R. H. Hak E. Rodenburg G. D. Bogaert D. Ijzerman E. P. (2009). Effect of reduced-dose schedules with 7-valent pneumococcal conjugate vaccine on nasopharyngeal pneumococcal carriage in children: a randomized controlled trial. *JAMA* 302 159–167. 10.1001/jama.2009.975 19584345

[B53] Vissers M. Wijmenga-Monsuur A. J. Knol M. J. Badoux P. van Houten M. A. van der Ende A. (2018). Increased carriage of non-vaccine serotypes with low invasive disease potential four years after switching to the 10-valent pneumococcal conjugate vaccine in The Netherlands. *PLoS One* 13:e0194823. 10.1371/journal.pone.0194823 29601605 PMC5877862

[B54] Watt J. P. O’Brien K. L. Katz S. Bronsdon M. A. Elliott J. Dallas J. (2004). Nasopharyngeal versus oropharyngeal sampling for detection of pneumococcal carriage in adults. *J. Clin. Microbiol.* 42 4974–4976. 10.1128/JCM.42.11.4974-4976.2004 15528682 PMC525247

[B55] Weinberger D. M. Grant L. R. Weatherholtz R. C. Warren J. L. O’Brien K. L. Hammitt L. L. (2016). Relating pneumococcal carriage among children to disease rates among adults before and after the introduction of conjugate vaccines. *Am. J. Epidemiol.* 183 1055–1062. 10.1093/aje/kwv283 27188949 PMC4887577

[B56] Weinberger D. M. Malley R. Lipsitch M. (2011). Serotype replacement in disease after pneumococcal vaccination. *Lancet* 378 1962–1973. 10.1016/S0140-6736(10)62225-8 21492929 PMC3256741

[B57] Welte T. Torres A. Nathwani D. (2012). Clinical and economic burden of community-acquired pneumonia among adults in Europe. *Thorax* 67 71–79. 10.1136/thx.2009.129502 20729232

[B58] Whalan R. H. Funnell S. G. Bowler L. D. Hudson M. J. Robinson A. Dowson C. G. (2006). Distribution and genetic diversity of the ABC transporter lipoproteins PiuA and PiaA within *Streptococcus pneumoniae* and related streptococci. *J. Bacteriol.* 188 1031–1038. 10.1128/JB.188.3.1031-1038.2006 16428407 PMC1347328

[B59] World Health Organization (2019). Pneumococcal conjugate vaccines in infants and children under 5 years of age: WHO position paper –February 2019 - Vaccins antipneumococciques conjugués chez les nourrissons et les enfants de moins de 5 ans: note de synthèse de l’OMS – février 2019. *Wkly. Epidemiol. Rec.* 94 85–103.

[B60] Wyllie A. L. Chu M. L. Schellens M. H. van Engelsdorp Gastelaars J. Jansen M. D. van der Ende A. (2014). *Streptococcus pneumoniae* in saliva of Dutch primary school children. *PLoS One* 9:e102045. 10.1371/journal.pone.0102045 25013895 PMC4094488

[B61] Wyllie A. L. Pannekoek Y. Bovenkerk S. van Engelsdorp Gastelaars J. Ferwerda B. van de Beek D. (2017). Sequencing of the variable region of rpsB to discriminate between *Streptococcus pneumoniae* and other streptococcal species. *Open Biol.* 7:170074. 10.1098/rsob.170074 28931649 PMC5627049

[B62] Wyllie A. L. Rumke L. W. Arp K. Bosch A. A. Bruin J. P. Rots N. Y. (2016a). Molecular surveillance on *Streptococcus pneumoniae* carriage in non-elderly adults; little evidence for pneumococcal circulation independent from the reservoir in children. *Sci. Rep.* 6:34888. 10.1038/srep34888 27713565 PMC5054371

[B63] Wyllie A. L. Wijmenga-Monsuur A. J. van Houten M. A. Bosch A. Groot J. A. van Engelsdorp Gastelaars J. (2016). Molecular surveillance of nasopharyngeal carriage of *Streptococcus pneumoniae* in children vaccinated with conjugated polysaccharide pneumococcal vaccines. *Sci. Rep.* 6:23809. 10.1038/srep23809 27046258 PMC4820691

[B64] Wyllie A. L. Warren J. L. Regev-Yochay G. Givon-Lavi N. Dagan R. Weinberger D. M. (2020). Serotype patterns of pneumococcal disease in adults are correlated with carriage patterns in older children. *Clin. Infect. Dis.* 72 e768–e775. 10.1093/cid/ciaa1480 32989457 PMC8315131

[B65] York A. Mbodj S. Yolda-Carr D. Hislop M. Weinberger D. M. Wyllie A. L. (2021). Magnetic Bead-Based Separation (MBS) of pneumococcal serotypes. *bioRxiv* [Preprint]. 10.1101/2021.06.06.447277 PMC10014298 36936076

