# Supplementary material for: It Takes Two to Tango: Combining Conventional Culture With Molecular Diagnostics Enhances Accuracy of Streptococcus pneumoniae Detection and Pneumococcal Serogroup/Serotype Determination in Carriage
Source: Front Microbiol. 2022 Apr 18;13:859736. doi: 10.3389/fmicb.2022.859736 (PMC9060910; doi:10.3389/fmicb.2022.859736)
Supplement: Supplementary file 1 [file Table_1.docx]

**Supplementary Table S1. qPCR programmes used in this study.**

| **qPCR assay** | **Step** | **Cycles** | **Temperature (°C)** | **Duration** |
| --- | --- | --- | --- | --- |
| *lytA, piaB,* all serotype/serogroup-specific assays except 17F | Pre-incubation | 1 | 95 | 10 min |
|  | Denaturation | 45 | 95 | 10 sec |
|  | Annealing & elongation |  | 60 | 45 sec |
| 17F | Pre-incubation | 1 | 95 | 10 min |
|  | Denaturation | 45 | 95 | 15 sec |
|  | Annealing & elongation |  | 60 | 3 min |
