# Supplementary material for: It Takes Two to Tango: Combining Conventional Culture With Molecular Diagnostics Enhances Accuracy of Streptococcus pneumoniae Detection and Pneumococcal Serogroup/Serotype Determination in Carriage
Source: Front Microbiol. 2022 Apr 18;13:859736. doi: 10.3389/fmicb.2022.859736 (PMC9060910; doi:10.3389/fmicb.2022.859736)
Supplement: Supplementary file 2 [file Table_2.docx]

**Supplementary Table S2. Primers and probes used in this study**

| **Serotype/serogroup** | **Mastermix** | **Primer/probe** | **Composition^1^** | **Concentration in PCR reaction (nM)** | **Reference** |
| --- | --- | --- | --- | --- | --- |
| 1 | TaqMan Universal | 1-Fw | 5’-CGTGCGGTAATTGAAGCTATGA-3’ | 200 | Azzari et al. 2010 |
|  |  | 1-Rv | 5’-TGTGGCCCCAGCAACTCT-3’ | 200 |  |
|  |  | 1-P | 5’-TGCTTGCCCTTGTATAGGGT-3’ | 200 |  |
| 3 | SensiFast | 3-Fw | 5’-GGTCAGCAGAAAGTATGCATTGG-3’ | 200 | Azzari et al. 2010 |
|  |  | 3-Rv | 5’-TCGTTTATCCAGGGTCTGATGA-3’ | 250 |  |
|  |  | 3-P | 5’-TATTGGATGTGGTTTATCGTGAAGA-3’ | 175 |  |
| 4 | SensiFast | 4-Fw | 5’-TGGGATGACATTTCTACGCACTA-3’ | 200 | Azzari et al. 2010 |
|  |  | 4-Rv | 5’-CCGTCGCTGATGCTTTATCA-3’ | 200 |  |
|  |  | 4-P | 5’-TCCTATTGGATGGTTAGTTGGTGA-3’ | 200 |  |
| 5 | SensiFast | 5-Fw | 5’-TTACGGGAGTATCTTATGTCTTTAATGG-3’ | 200 | Azzari et al. 2010 |
|  |  | 5-Rv | 5’-CAGCATTCCAGTAGCCTAAAACTAGA-3’ | 200 |  |
|  |  | 5-P | 5’-TTGTCTCAGCAACTCTATTTGGCTGTGGG-3’ | 200 |  |
| 6A/B/C/D | TaqMan Universal | 6ABCD-Fw | 5’-AAGTTTGCACTAGAGTATGGGAAGGT-3’ | 200 | Azzari et al. 2010 |
|  |  | 6ABCD-Rv | 5’-ACATTATGTCCRTGTCTTCGATACAAG-3’ | 200 |  |
|  |  | 6ABCD-P | 5’-TGTTCTGCCCTGAGCAACTGG-3’ | 125 |  |
| 7A/F | SensiFast | 7AF-Fw | 5’-GATGGCATGTGGCAAACCA-3’ | 250 | Azzari et al. 2010 |
|  |  | 7AF-Rv | 5’-TTTGCCCTCCTTAATCATTTCAC-3’ | 200 |  |
|  |  | 7AF-P | 5’-TTGGCTATCGGCATGGTGGT-3’ | 175 |  |
| 8 | SensiFast | 8-Fw | 5’-CCACTCATCAGTTTCCCATATGTTT-3’ | 250 | Azzari et al. 2010 |
|  |  | 8-Rv | 5’-TCAATAATTGAAGAAGCGAACGTT-3’ | 250 |  |
|  |  | 8-P | 5’-TGATGGCAGATGGGTTGGGACGAG-3’ | 175 |  |
| 9A/L/N/V | SensiFast | 9AV-Fw | 5’-TGGAATGGGCAAAGGGTAGTA-3’ | 200 | Azzari et al. 2010 |
|  |  | 9AV-Rv | 5’-TCGGTTCCCCAAGATTTTCTC-3’ | 250 |  |
|  |  | 9AV-P | 5’-TTAATCATGCTAACGGCTCATCGA-3’ | 175 |  |
| 10A/B | SensiFast | 10AB-Fw | 5’-CCTCTCCTATCAACTATTACTCATTATACTACCT-3’ | 250 | Azzari et al. 2010 |
|  |  | 10AB-Rv | 5’-AATAACCATAAGTCCCTAGATCATTCAAAG-3’ | 250 |  |
|  |  | 10AB-P | 5’-TCATTACAACTCCCTATGTGACACGGGTCTTTT-3’ | 175 |  |
| 11A/D | SensiFast | 11AD-Fw | 5’-AAATGGTTTGGATATGGTTTGTTTGG-3’ | 150 | Pimenta et al. 2013 |
|  |  | 11AD-Rv | 5’-AGTGCTAACTGTAAAACTTGATTATGAG-3’ | 200 |  |
|  |  | 11AD-P | 6-FAM 5’-ATTCCAACTTCTCCCAATTTCTGCCACGG-3’ BHQ-1 | 175 |  |
| 12A/B/F | SensiFast | 12ABF-Fw | 5’-GATTATTCGCTTGCCTCTTCATG-3’ | 250 | Azzari et al. 2010 |
|  |  | 12ABF-Rv | 5’-ATAGCCGAAATAAGCTTTCCAGAA-3’ | 250 |  |
|  |  | 12ABF-P | 5’-ATTTGTAAGCGGACGTGCGATT-3’ | 200 |  |
| 14 | SensiFast | 14-Fw | 5’-CGACTGAAATGTCACTAGGAGAAGAT-3’ | 250 | Azzari et al. 2010 |
|  |  | 14-Rv | 5’-AATACAGTCCATCAATTACTGCAATACTC-3’ | 250 |  |
|  |  | 14-P | 6-FAM 5’-TGTCATTCGTTTGCCAATACTTGATGGTCTC-3’ BHQ-1 | 150 |  |
| 15A/B/C/F | SensiFast | 15-Fw | 5’-TTGAATCAGGTAGATTGATTTCTGCTA-3’ | 250 | Azzari et al 2010 |
|  |  | 15-Rv | 5’-CTCTAGGAATCAAATACTGAGTCCTAATGA-3’ | 250 |  |
|  |  | 15-P | 6-FAM 5’-CTCCGGCTTTTGTCTTCTCTGT-3’ BHQ-1 | 175 |  |
| 16F | TaqMan Universal | 16F-Fw | 5’-CAGGCGAAAAGCGAGCAT-3’ | 300 | Azzari et al. 2012 |
|  |  | 16F-Rv | 5’-TGGGTTCCCCTCATCTACGTT-3’ | 300 |  |
|  |  | 16F-P | 5’-TGCTTTGGTAGCTTGTATGAGTGC-3’ | 200 |  |
| 17F | SensiFast | 17F-Fw | 5’-CTTAGCGTACGTTCTTCGTATGCTA-3’ | 400 | Azzari et al. 2012 |
|  |  | 17F-Rv | 5’-CCCGTACTCGGAAGCAAAAC-3’ | 400 |  |
|  |  | 17F-P | 6-FAM 5’-TCTAAGAGAGCTACTGAAACACTTTGTGC-3’ BHQ-1 | 200 |  |
| 18A/B/C/F | SensiFast | 18ABCF-Fw | 5’-TCGATGGCTAGAACAGATTTATGG-3’ | 150 | Pimenta et al. 2013 |
|  |  | 18ABCF-Rv | 5’-CCATTGTCCCTGTAAGACCATTG-3’ | 150 |  |
|  |  | 18ABCF-P | 6-FAM 5’-AGGGAGTTGAATCAACCTATAATTTCGCCCC-3’ BHQ-1 | 150 |  |
| 19A | SensiFast | 19A-Fw | 5’-TTCGACGACGTATCAGCTTCA-3’ | 200 | Azzari et al. 2010 |
|  |  | 19A-Rv | 5’-TCATTGAGAGCCTTAACCTCTTCA-3’ | 200 |  |
|  |  | 19A-P | 5’-ACCCAAAACGGTTGACGCATTATACT-3’ | 200 |  |
| 19F | SensiFast | 19F-Fw | 5’-TGAGGTTAAGATTGCTGATCG-3’ | 300 | Pimenta et al. 2013 |
|  |  | 19F-Rv | 5’-CACGAATGAGAACTCGAATAAAAG-3’ | 300 |  |
|  |  | 19F-P^2^ | Cy5 5’-CGCACTGTCAATTCACCTTC-3’ BHQ-2 | 100 |  |
| 20 | SensiFast | 20-Fw | 5’-AAAGATACTGGCTGAGGAGCTATCTATT-3’ | 150 | Azzari et al. 2010 |
|  |  | 20-Rv | 5’-AGTCAAAAGTACTCAACCATTCTGATATATTC-3’ | 200 |  |
|  |  | 20-P | 5’-AGGATAAGGTCTACTTTGTGGGAGTTC-3’ | 200 |  |
| 21 | SensiFast | 21-Fw | 5’-GGTTTAAATATCGCTCCGGGTAT-3’ | 400 | Azzari et al. 2012 |
|  |  | 21-Rv | 5’-CAAAAAAAGGGCTTGTAGACGAA-3’ | 400 |  |
|  |  | 21-P | 5’-TGTGAATTGGACACGTTATGGAGC-3’ | 200 |  |
| 22A/F | SensiFast | 22AF-Fw | 5’-TCTATTAAATAACCCATTGGAATTGAAACG-3’ | 250 | Pimenta et al. 2013^#^ |
|  |  | 22AF-Rv | 5’-TCGCAATTGAAGACCACATAAACTG-3’ | 250 |  |
|  |  | 22AF-P^#^ | 5’-TCCGTAATGCGCTTATGAGCACATTCTCCA-3’ | 150 |  |
| 23A | SensiFast | 23A-Fw | 5’-GGGAATTTGGCACTCTTCTGAAT-3’ | 400 | Azzari et al. 2012 |
|  |  | 23A-Rv | 5’-GATCGGCAAATGTTGAAACCA-3’ | 400 |  |
|  |  | 23A-P | 5’-TTGGCGGTAAACAATTAAGGCGT-3’ | 200 |  |
| 23B | SensiFast | 23B-Fw | 5’-TTGAAGAAATTGCTCCAGAAACAT-3’ | 400 | Azzari et al. 2012 |
|  |  | 23B-Rv | 5’-CCAAAAGACTAGCCTCAACCACTAA-3’ | 400 |  |
|  |  | 23B-P | 5’-TAGAGCTATTTATCTTTCGTGGTTTT-3’ | 200 |  |
| 23F | SensiFast | 23F-Fw | 5’-TGCTATTTGCGATCCTGTTCAT-3’ | 250 | Azzari et al. 2010 |
|  |  | 23F-Rv | 5’-AGAGCCTCCGTTGTTTCGTAAA-3’ | 150 |  |
|  |  | 23F-P | 5’-TTTCTCCGGCATCAAACGTTAAG-3’ | 175 |  |
| 33A/F/37 | SensiFast | 33AF-Fw | 5’-CGAGAGAGAATATGAGGGAATTGTTA-3’ | 250 | Azzari et al. 2010 |
|  |  | 33AF-Rv | 5’-TCTCAATCCCCGCATTTACTG-3’ | 200 |  |
|  |  | 33AF-P | 6-FAM 5’-AGGAAAACTGTGGTCACGGTTCG-3’ BHQ-1 | 125 |  |
| 34 | SensiFast | 34-Fw | 5’-CGGTGGAGTAGGTCAAGATG-3’ | 400 | Sakai et al. 2015 |
|  |  | 34-Rv | 5’-GTCTGTTCTCCCCAATATACTGAG-3’ | 400 |  |
|  |  | 34-P | 5’-ACGGAGCGCCAATGTACTTGAATAGTT-3’ | 200 |  |
| 35B/C | SensiFast | 35BC-Fw | 5’-GCATGGAGGTGGAGCATACA-3’ | 200 | Azzari et al. 2010 |
|  |  | 35BC-Rv | 5’-TGTAAAGACTGCACAACTCGATATAAAA-3’ | 250 |  |
|  |  | 35BC-P | 5’-CAATTTAAACAATATTAGTAAAGCGCAGGTCAAGCAAA-3’ | 125 |  |
| 38 | SensiFast | 38-Fw | 5’-GTCTTACGTAGAACCTCTCTGGATGA-3’ | 200 | Azzari et al. 2010 |
|  |  | 38-Rv | 5’-TGGTCCTACAAGCGACATGTG-3’ | 200 |  |
|  |  | 38-P | 5’-TTGCCACAGATTTGGAATATTTTGGTCGG-3’ | 150 |  |

Fw: forward primer, Rv: reverse primer, P: probe, SensiFast : SensiFast Probe No-ROX mix (Bioline), TaqMan Universal PCR master mix (ThermoFisher Scientific).^1^Unless marked otherwise, all probes with 6-FAM as reporter at 5’ and TAMRA as quencher at 3’ end. ^2^ Locked nucleic acid nucleotides are underlined.# : The 22A/F probe sequence was adapted from Pimenta et al. 2013 to detect a variant circulating in the Netherlands.
