# Supplementary material for: It Takes Two to Tango: Combining Conventional Culture With Molecular Diagnostics Enhances Accuracy of Streptococcus pneumoniae Detection and Pneumococcal Serogroup/Serotype Determination in Carriage
Source: Front Microbiol. 2022 Apr 18;13:859736. doi: 10.3389/fmicb.2022.859736 (PMC9060910; doi:10.3389/fmicb.2022.859736)
Supplement: Supplementary file 3 [file Table_3.docx]

**Supplementary Table S3.** Results of an intraclass correlation coefficient (ICC) reliability analysis for ^ROCd^C_q_-based criteria in a subset of samples classified as positive for *S. pneumoniae* according to the ^A^C_q_ criterium of <40.

| **Sample** | **C*q* score** | **Number of samples** | **ICC**  ***(95% CI)*** | ***p*-value** |
| --- | --- | --- | --- | --- |
| Minimally processed nasopharyngeal | < ^ROCd^C_q_ | 512 | 0.96  *(0.94-0.97)* | < 0.0001 |
|  | > ^ROCd^C_q_ | 49 | -0.15  *(-0.42-0.14)* | 0.845 |
| Culture-enriched nasopharyngeal | < ^ROCd^C_q_ | 469 | 0.92  *(0.73-0.97)* | < 0.0001 |
|  | > ^ROCd^C_q_ | 10 | -0.19  *(-0.59-0.41)* | 0.751 |
| Minimally processed oropharyngeal | < ^ROCd^C_q_ | 59 | 0.96  *(0.93-0.97)* | < 0.0001 |
|  | > ^ROCd^C_q_ | 24 | -0.323  *(-0.62-0.08)* | 0.947 |
| Culture-enriched oropharyngeal | < ^ROCd^C_q_ | 39 | 0.99  *(0.99-1)* | < 0.0001 |
|  | > ^ROCd^C_q_ | 19 | 0.31  *(-0.19-0.66)* | 0.104 |

ICC: intraclass correlation coefficient - a single score intraclass correlation coefficient with two-way model was used for reliability analysis. ICC values <0.50, 0.50-0.75, 0.75-0.90 and >0.90 are indicative of poor, moderate, good, and excellent reliability, respectively. ^ROCd^C_q_: Receiver operating characteristic (ROC) curve analysis-derived Cq cut-off value, ^A^C_q_: arbitrary criterium-derived cut-off value.
