# Supplementary material for: It Takes Two to Tango: Combining Conventional Culture With Molecular Diagnostics Enhances Accuracy of Streptococcus pneumoniae Detection and Pneumococcal Serogroup/Serotype Determination in Carriage
Source: Front Microbiol. 2022 Apr 18;13:859736. doi: 10.3389/fmicb.2022.859736 (PMC9060910; doi:10.3389/fmicb.2022.859736)
Supplement: Supplementary file 4 [file Table_4.docx]

**Supplementary Table S4**. The accuracy of *Streptococcus pneumoniae* qPCR detection in n=1549 nasopharyngeal samples from children (n=946) and adults (n=603), and n=319 oropharyngeal samples from adults tested using molecular methods applied to DNA extracted from culture-enriched samples and applying ^ROCd^C_q_ or ^A^C_q_ thresholds for a sample positivity in qPCRs with either the ‘two-to-tango’ approach (*lytA* & *piaB*) or based on *piaB* or *lytA* results alone. Measures of diagnostic accuracy were calculated by comparing the number of detected samples positive per method with the number of individuals positive for *S. pneumoniae* based on isolation of live pneumococcus either from the primary diagnostic or qPCR-guided culture.

| **Method** | **Study population** | **Assay** | **Percent (n) of positive samples**  ***(95%CI)*** | **PPV**  **%**  **(*95%CI*)** | **NPV**  **%**  **(*95%CI*)** | **Sensitivity %**  **(*95%CI*)** | **Specificity  %**  **(*95%CI*)** | **Concordance %**  **(*95%CI*)** | ***κ***  **(*95%CI*)** |
| --- | --- | --- | --- | --- | --- | --- | --- | --- | --- |
| qPCRs on culture-enriched  nasopharyngeal samples | All | *piaB & lytA,* ^ROCd^C_q_ | 30.3 (469)  *(28.0 – 32.6)* | 95.7  *(93.5 – 97.2)* | 99.0  *(98.2 – 99.4)* | 97.6  *(95.8 – 98.7)* | 98.2  *(97.2 – 98.8)* | 98.0  *(97.2 – 98.6)* | 0.95  *(0.94 – 0.97)* |
|  |  | *piaB,* ^ROCd^C_q_ | 30.5 (473)  *(28.3 – 32.9)* | 95.3  *(93.1 – 96.9)* | 99.2  *(98.4 – 99.6)* | 98.0  *(96.3 – 99.0)* | *98.0*  *(97.0 -98.7)* | 98.0  *(97.2 – 98.6)* | *0.95*  *(0.94 – 0.97)* |
|  |  | *lytA,* ^ROCd^C_q_ | 31.6 (490)  *(29.4 – 34.0)* | 91.8  *(89.1 – 93.9)* | 99.1  *(98.3 – 99.5)* | 97.8  *(96.0 – 98.8)* | 96.3  *(95.0 – 97.3)* | 96.8  *(95.0 – 97.3)* | 0.92  *(0.90 – 0.94)* |
|  |  | *piaB & lytA,* ^A^C_q_ | 30.9 (479)  *(28.7 – 33.3)* | 94.4  *(91.9 – 96.1)* | 99.3  *(98.5 – 99.6)* | 98.3  *(96.6 – 99.1)* | 97.5  *(96.4 – 98.3)* | 97.7  *(96.9 – 98.4)* | 0.95  *(0.93 – 0.96)* |
|  |  | *piaB,* ^A^C_q_ | 31.3 (485)  *(29.0 – 33.7)* | 93.2  *(90.6 – 95.1)* | 99.2  *(98.5 – 99.6)* | 98.3  *(96.6 – 99.1)* | 97.0  *(95.8 – 97.8)* | 97.4  *(96.4 – 98.0)* | 0.94  *(0.92 – 0.96)* |
|  |  | *lytA,* ^A^C_q_ | 32.5 (503)  *(30.2 – 34.8)* | 89.9  *(86.9 – 92.2)* | 99.2  *(98.5 – 99.6)* | 98.3  *(96.6 – 99.1)* | 95.3  *(93.9 – 96.4)* | 96.2  *(95.1 – 97.0)* | 0.91  *(0.89 – 0.93)* |
| qPCRs on culture-enriched  oropharyngeal samples | Adult | *piaB & lytA,* ^ROCd^C_q_ | 12.2 (39)  *(9.1 – 16.3)* | 59.0  *(43.4 – 72.9)* | 100  *(98.6 – 100)* | 100  *(85.7 – 100)* | 94.3  *(91.0 – 96.4)* | 95.0  *(92.0 – 96.9)* | 0.72  *(0.58 – 0.85)* |
|  |  | *piaB,* ^ROCd^C_q_ | 12.5 (40)  *(9.3 – 16.6)* | 57.5  *(42.2 – 71.5)* | 100  *(98.6 – 100)* | 100  *(85.7 – 100)* | 94.3  *(91.0 – 96.4)* | 94.7  *(91.6 – 96.6)* | 0.70  (0.57 – 0.84) |
|  |  | *lytA,* ^ROCd^C_q_ | 13.2 (42)  *(9.9 – 17.3)* | 54.8  *(39.9 – 68.8)* | 100  *(98.6 – 100)* | 100  *(85.7 – 100)* | 93.6  *(90.2 – 95.9)* | 94.0  *(90.9 – 96.2)* | 0.68  *(0.54 – 0.82)* |
|  |  | *piaB & lytA,* ^A^C_q_ | 18.2 (58)  *(14-3 – 22.8)* | 39.7  *(28.1 – 52.5)* | 100  *(98.5 – 100)* | 100  *(85.7 – 100)* | 88.2  *(84.0 – 91.4)* | 89.0  *(85.1 – 92.0)* | 0.52  *(0.32 – 0.67)* |
|  |  | *piaB,* ^A^C_q_ | 18.8 (60)  *(14.9 – 23.5)* | 38.3  *(27.1 – 51.0)* | 100  *(98.5 – 100)* | 100  *(85.7 – 100)* | 87.5  *(83.2 – 90.8)* | 88.4  *(84.4 – 91.5)* | 0.50  *(0.35 – 0.65)* |
|  |  | *lytA,* ^A^C_q_ | 30.3 (96)  *(25.3 – 35.3)* | 24  *(16.5 – 33.4)* | 100  *(98.3 – 100)* | 100  *(85.7 – 100)* | 75.3  *(70.1 – 79.9)* | 77.1  *(72.2 – 81.4)* | 0.31  *(0.17 – 0.45)* |

^ROCd^C_q_ – receiver operating characteristic curve derived Cq cut-off value with culture plus reculture as reference; ^A^C_q_ – arbitrary Cq cut-off value of <40 Cq; PPV – positive predictive value; NPV – negative predictive value; 95%CI – 95% confidence interval; *κ* – Cohen’s kappa where ≤0, 0.01-0.20, 0.21-0.40, 0.41-0.60, 0.61-0.80, >0.81 are interpreted as poor agreement, slight, fair, moderate, substantial, and almost perfect agreement, respectively.
