# Supplementary material for: It Takes Two to Tango: Combining Conventional Culture With Molecular Diagnostics Enhances Accuracy of Streptococcus pneumoniae Detection and Pneumococcal Serogroup/Serotype Determination in Carriage
Source: Front Microbiol. 2022 Apr 18;13:859736. doi: 10.3389/fmicb.2022.859736 (PMC9060910; doi:10.3389/fmicb.2022.859736)
Supplement: Supplementary file 5 [file Table_5.docx]

**Supplementary Table S5. Number of serotypes/serogroups detected with any method.**

|  | **Nasopharyngeal samples from children** | | | **Nasopharyngeal samples from adults** | | |
| --- | --- | --- | --- | --- | --- | --- |
| **number of detected serotypes** | **by any method**  **n (%)** | **by culture**  **n (%)** | **by qPCR***  **n (%)** | **by any method**  **n (%)** | **by culture**  **n (%)** | **by qPCR* n (%)** |
| 0 | 543 (57.4) | 524 (55.4) | 551 (58.2) | 583 (96.7) | 580 (96.2) | 587 (97.3) |
| 1^#^ | 357 (37.7) | 415 (43.9) | 352 (37.2) | 19 (3.2) | 23 (3.8) | 15 (2.5) |
| 2 | 40 (4.2) | 7 (0.7) | 37 (3.9) | 1 (0.2) | 0 (-) | 1 (0.2) |
| 3 | 4 (0.4) | 0 (-) | 4 (0.4) | 0 (-) | 0 (-) | 0 (-) |
| 4 | 1 (0.1) | 0 (-) | 1 (0.1) | 0 (-) | 0 (-) | 0 (-) |
| 5 | 1 (0.1) | 0 (-) | 1 (0.1) | 0 (-) | 0 (-) | 0 (-) |

^#^ : includes samples that are pneumococcus-positive in qPCR with no signal for any serotype. *: qPCR detection was performed after culture-enrichment of the nasopharyngeal sample.
