# Supplementary material for: It Takes Two to Tango: Combining Conventional Culture With Molecular Diagnostics Enhances Accuracy of Streptococcus pneumoniae Detection and Pneumococcal Serogroup/Serotype Determination in Carriage
Source: Front Microbiol. 2022 Apr 18;13:859736. doi: 10.3389/fmicb.2022.859736 (PMC9060910; doi:10.3389/fmicb.2022.859736)
Supplement: Supplementary file 6 [file Table_6.docx]

**Supplementary Table S6.** Optimal qPCR cycle threshold C_q_ and corresponding parameters for *Streptococcus pneumoniae* carriage detection in a subset of nasopharyngeal (n=176) and oropharyngeal (n=100) samples processed independently in England and in the Netherlands. Results from qPCR were validated in a receiver operating characteristic (ROC) curve analysis with culture as reference*.

| **Sample** | **Target** | **ICC**  (*95% CI*) | **England** | | | | **Netherlands** | | | |
| --- | --- | --- | --- | --- | --- | --- | --- | --- | --- | --- |
|  |  |  | **Optimal threshold**  (*95% CI*) | **Youden index** | **Sensitivity** | **Specificity** | **Optimal**  **threshold**  (*95% CI*) | **Youden**  **index** | **Sensitivity** | **Specificity** |
| MP NP | *piaB* | 0.76  *(0.64 – 0.84)* | 37.85  *(35.05 – 39.25)* | 0.66 | 0.81 | 0.85 | 36.99  *(35.92 – 37.44)* | 0.94 | 0.96 | 0.98 |
| MP NP | *lytA* | 0.79  *(0.69 – 0.85)* | 36.77  *(34.33 – 37.34)* | 0.66 | 0.78 | 0.88 | 35.17  *(32.6 – 36.74)* | 0.73 | 0.85 | 0.88 |
| CE NP | *piaB* | 0.82  *(0.56 – 0.91)* | 24.30  *(21.61 – 28.87)* | 0.86 | 0.95 | 0.91 | 27.51  *(24.12 – 29.26)* | 0.96 | 0.99 | 0.97 |
| CE NP | *lytA* | 0.86  *(0.52 – 0.94)* | 26.51  *(22.09 – 30.01)* | 0.81 | 0.96 | 0.85 | 25.47  *(24.36 – 27.93)* | 0.91 | 0.96 | 0.95 |
| CE OP | *piaB* | 0.78  *(0.62 – 0.87)* | 28.71  *(26.36 – 29.11)* | 0.75 | 0.89 | 0.86 | 29.43  *(26.72 – 29.64)* | 0.74 | 0.89 | 0.85 |
| CE OP | *lytA* | 0.87  *(0.81 – 0.91)* | 31.50  *(28.65 – 32.59)* | 0.77 | 0.89 | 0.88 | 30.28  *(27.50 – 30.77)* | 0.72 | 0.89 | 0.83 |

MP: minimally processed; CE: culture-enriched; NP: nasopharyngeal; OP: oropharyngeal. 95%CI – 95% confidence interval.

*Consents given by study participants in the Netherlands were not explicit about export of minimally processed samples outside the country. Consequently, detection of pneumococcus in minimally processed samples collected in the Netherlands could not be performed independently in England and results of culture (primary and qPCR-guided) in the country of samples origin, England for NP and the Netherlands for OP samples, were used as the references.
