# Supplementary material for: It Takes Two to Tango: Combining Conventional Culture With Molecular Diagnostics Enhances Accuracy of Streptococcus pneumoniae Detection and Pneumococcal Serogroup/Serotype Determination in Carriage
Source: Front Microbiol. 2022 Apr 18;13:859736. doi: 10.3389/fmicb.2022.859736 (PMC9060910; doi:10.3389/fmicb.2022.859736)
Supplement: Supplementary file 7 [file Table_7.docx]

**Supplementary Table S7.** The accuracy of *Streptococcus pneumoniae* detection in n=176 nasopharyngeal (NP) and n=59 oropharyngeal (OP) samples tested independently in both countries using molecular methods applied to DNA extracted from minimally processed and culture-enriched samples and applying ^ROCd^C_q_ thresholds for a sample positivity in qPCRs. Measures of diagnostic accuracy were calculated by comparing the number of detected nasopharyngeal and oropharyngeal samples positive per method with the number of individuals positive for *S. pneumoniae* based on isolation of live pneumococcus either from the primary diagnostic or qPCR-guided culture (n=101 of nasopharyngeal samples culture-positive in UK and n=19 oropharyngeal samples culture-positive in the Netherlands).

| **Method** | **Percent (n) of samples tested positive in the Netherlands**  ***(95%CI)*** | **Percent (n) of samples tested positive in England**  ***(95%CI)*** | **Concordance %**  **(*95%CI*)** | ***κ***  **(*95%CI*)** |
| --- | --- | --- | --- | --- |
| qPCRs on minimally processed NP samples | 42.0 (74)  *(35.0 – 49.4)* | 54.5 (96)  *(47.2 – 61.7)* | 83.0  *(76.7 – 87.8)* | 0.66  *(0.55 – 0.77)* |
| qPCRs on culture-enriched NP samples | 59.1 (104)  *(51.7 – 66.1)* | 55.1 (97)  *(47.7 – 62.3)* | 93.8  *(89.2 – 96.5)* | 0.87  *(0.80 – 0.95)* |
| qPCRs on culture-enriched OP samples | 22.0 (13)  *(13.4 – 34.1)* | 25.4 (15)  *(16.1 – 37.8)* | 98.0  *(93.0 – 99.4)* | 0.95  *(0.88 - 1)* |

95%CI – 95% confidence interval; *κ* – Cohen’s Kappa where ≤0, 0.01-0.20, 0.21-0.40, 0.41-0.60, 0.61-0.80, >0.81 are interpreted as no agreement, none to slight, fair, moderate, substantial, and almost perfect agreement, respectively.
