# Supplementary figures and images for: It Takes Two to Tango: Combining Conventional Culture With Molecular Diagnostics Enhances Accuracy of Streptococcus pneumoniae Detection and Pneumococcal Serogroup/Serotype Determination in Carriage
Source: Front Microbiol. 2022 Apr 18;13:859736. doi: 10.3389/fmicb.2022.859736 (PMC9060910; doi:10.3389/fmicb.2022.859736)

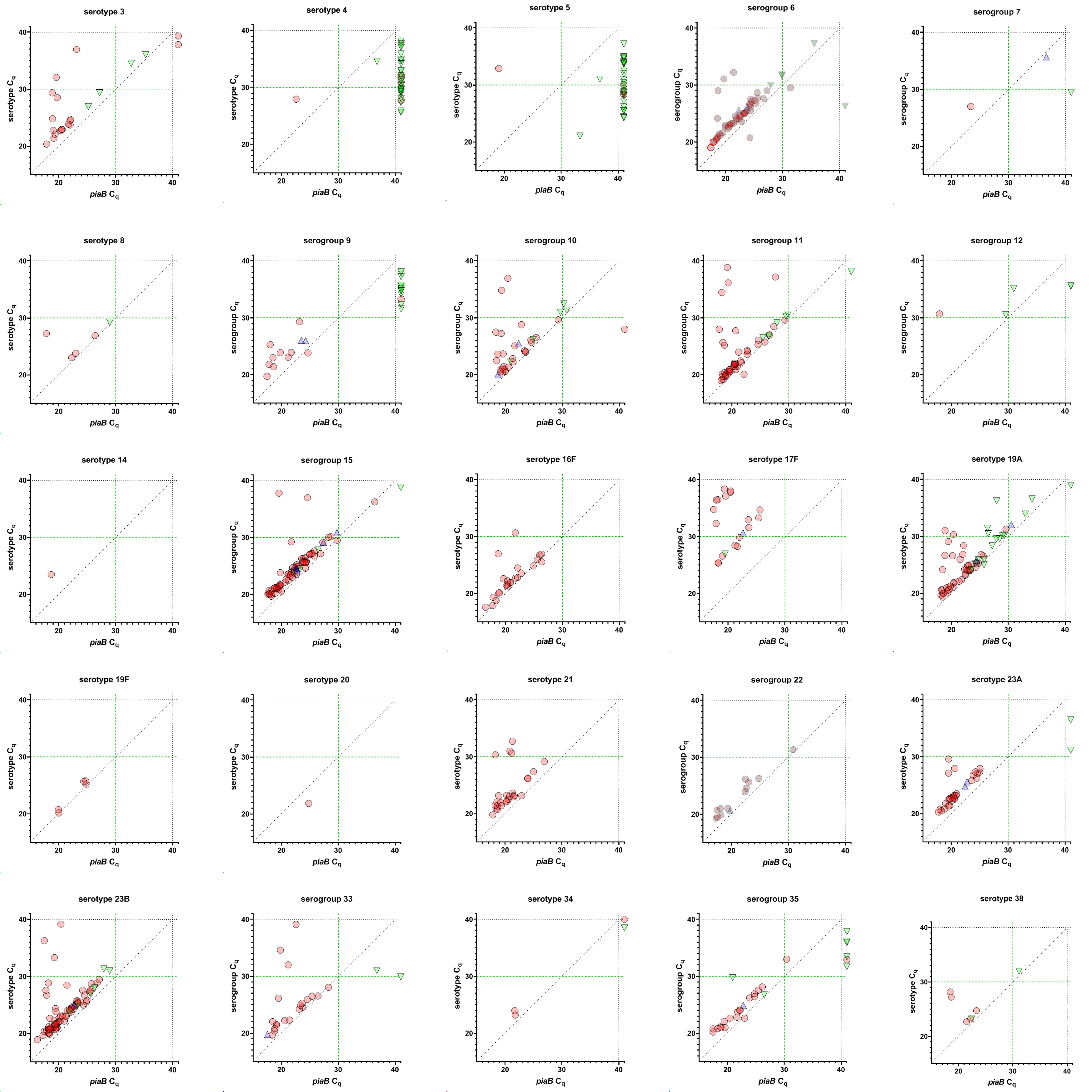

Supplement: Supplementary Figure 1 — Results of molecular method-based quantification of serogroups/serotypes-specific sequences in DNA from culture-enriched nasopharyngeal and oropharyngeal samples. Individual scatter plots depict results of a single serotype-specific or serogroup-specific qPCR assay as labeled above the panel. Each symbol represents an individual sample: pale-red dots represent nasopharyngeal samples from children; blue triangles and green triangles represent nasopharyngeal and oropharyngeal samples from adults, respectively. Sample was classified as positive for a serotype/serogroup with molecular method when the signal detected by qPCR for piaB (X-axis) and serotype/serogroup (Y-axis) were both below the ROCdCq cut-off threshold of 30 Cq. Only samples that generated a signal of Cq < 40 in a particular serogroup/serotype-specific qPCR are depicted. Symbols of Cq > 40 for piaB depict individual samples or pools of samples negative for pneumococcus by qPCR. In the panel depicting qPCR results for serogroup 6 all symbols represent samples identified as positive for serotypes 6C or 6D except for red circles depicting two samples identified as positive for serotype 6A or 6B, both positive for serotype 6A by culture. In the panel depicting qPCR results for serogroup 22 all symbols represent positivity for serotype 22F. None of the samples generated any signal in qPCRs targeting serotype 1, serotype 23F and serogroup 18. In addition, no samples were identified as positive for serogroup 12 according to study criteria. [file Image_1.tif]

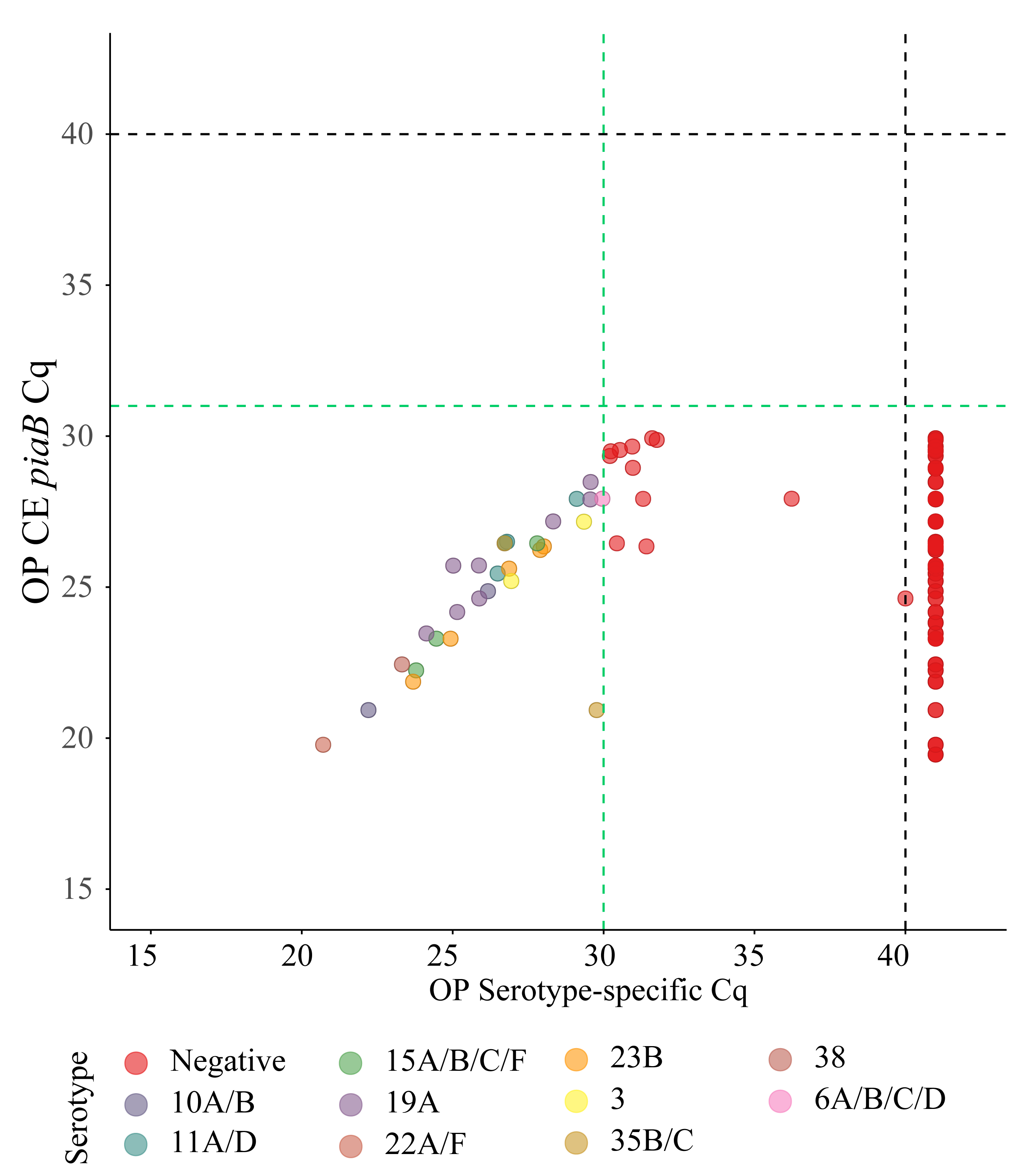

Supplement: Supplementary Figure 2 — Scatter plot displaying correlation between cycle threshold (Cq) from real-time PCR (qPCR) assays targeting the Streptococcus pneumoniae piaB and serotype/serogroup specific signal detected with qPCR for culture-enriched (CE) oropharyngeal samples from adults classified positive for S. pneumoniae according to ROCdCq criterium (green dashed lines). Dots depicts serotypes detected with qPCR. Dots are color-coded according to serotypes/serogroup targeted in an assay (see legend). [file Image_2.tif]
